# Integrative multi-omics and drug–response characterization of patient-derived prostate cancer primary cells

**DOI:** 10.1038/s41392-023-01393-9

**Published:** 2023-05-01

**Authors:** Ziruoyu Wang, Yanan Li, Wensi Zhao, Shuai Jiang, Yuqi Huang, Jun Hou, Xuelu Zhang, Zhaoyu Zhai, Chen Yang, Jiaqi Wang, Jiying Zhu, Jianbo Pan, Wei Jiang, Zengxia Li, Mingliang Ye, Minjia Tan, Haowen Jiang, Yongjun Dang

**Affiliations:** 1 Key Laboratory of Metabolism and Molecular Medicine, The Ministry of Education, Department of Biochemistry and Molecular Biology, School of Basic Medical Sciences, Shanghai Medical College, Fudan University, 200032 Shanghai, China; 2 CAS Key Lab of Separation Sciences for Analytical Chemistry, National Chromatographic Research and Analysis Center, Dalian Institute of Chemical Physics, Chinese Academy of Sciences, 116023 Dalian, China; 3 The Chemical Proteomics Center and State Key Laboratory of Drug Research, Shanghai Institute of Materia Medica, Chinese Academy of Sciences, 201203 Shanghai, China; 4 University of Chinese Academy of Sciences, 100049 Beijing, China; 5 Department of Urology, Zhongshan Hospital, Fudan University, 200032 Shanghai, China; 6 Department of Urology, Zhongshan Hospital Wusong Branch, Fudan University, 200032 Shanghai, China; 7 Center for Novel Target and Therapeutic Intervention, Chongqing Medical University, 400016 Chongqing, China; 8 Department of Urology, Huashan Hospital, Fudan University, 200040 Shanghai, China

**Keywords:** Bioinformatics, Urological cancer, Drug screening

## Abstract

Prostate cancer (PCa) is the second most prevalent malignancy in males across the world. A greater knowledge of the relationship between protein abundance and drug responses would benefit precision treatment for PCa. Herein, we establish 35 Chinese PCa primary cell models to capture specific characteristics among PCa patients, including gene mutations, mRNA/protein/surface protein distributions, and pharmaceutical responses. The multi-omics analyses identify Anterior Gradient 2 (AGR2) as a pre-operative prognostic biomarker in PCa. Through the drug library screening, we describe crizotinib as a selective compound for malignant PCa primary cells. We further perform the pharmacoproteome analysis and identify 14,372 significant protein-drug correlations. Surprisingly, the diminished AGR2 enhances the inhibition activity of crizotinib via ALK/c-MET-AKT axis activation which is validated by PC3 and xenograft model. Our integrated multi-omics approach yields a comprehensive understanding of PCa biomarkers and pharmacological responses, allowing for more precise diagnosis and therapies.

## Introduction

Prostate cancer (PCa) was the most frequently diagnosed cancer in men in over half (112 of 185) of the world’s countries in 2020.^1^ Throughout the past decade, systemic therapy for patients with Asia PCa has made very modest progress. Although only 7.8% of new cases of PCa worldwide occurred in China, 14.5% of deaths happened among Chinese patients according to GLOBOCAN 2020 estimates.^1^ One of the possible causes for higher mortality rate among Chinese PCa patients could be the unique genomic alteration signatures in Asian patients, such as gene-level mutation frequencies and copy number alternations, compared to those of Western populations, highlighting the importance of considering ethnic background when making clinical decisions.^2^

Currently, the early recognition of PCa and the prediction of tumor development following treatment still mainly relied on changes of prostate-specific antigen (PSA) level. However, 4–10 ng/mL PSA levels (also known as the gray area of PSA) cannot accurately distinguish benign prostatic hyperplasia (BPH) from malignancy or predict tumor progression. In addition, both BPHs and tumors have a phenotype of excessive cell growth, but only tumors exhibit a malignant phenotype of metastasis and invasion, the mechanism of which is not well understood. Consequently, investigating the molecular distinctions between BPH and tumor could improve our understanding of the roots of tumor malignancy.

In recent years, next-generation sequencing (NGS) has allowed for individualized cancer treatment.^3,4^ Nevertheless, accumulating evidence suggested that many of the cancer characteristics that influence tumor progression and treatment responses were influenced by non-genetic mechanisms,^5^ resulting in only 30% of patients exhibiting clinical responses commensurate with early expectations.^6,7^ Consequently, it is essential to utilize multi-level data to inform therapeutic decision making.

To date, limited PCa drug investigations in Asian populations have limited its clinical oncology efficacy. Directly exposing cells to drugs through functional precision medicine (FPM) could assist to identify dynamic individual critical vulnerabilities to improve the therapeutic efficacy in clinic.^8^ There is a growing interest in the pan-cancer platforms available for pharmaceutical screening based on widely used cell lines, including the Cancer Therapeutics Response Portal (CTRP),^9^ Genomics of Drug Sensitivity in Cancer (GDSC)^10^ and Cancer Cell Line Encyclopedia (CCLE).^11^ However, most of these were utilizing cell lines generated decades ago which may have lost their initial tumor features and developed new mutations as a result of long-term cultivation. Additionally, these cell lines were rarely derived from Asian population. To faithfully analyze personalized tumor heterogeneity and pharmaceutical responses, it is necessary to establish a panel of PCa patient-derived primary cell models and identify protein-drug associations.

The incidence of PCa in Asia is rapidly rising with economic development, longer life expectancy and adoption of the western lifestyle.^12,13^ Studies which integrated multi-omics and drug responses would be essential to improve treatment recommendations for Asian male populations. Herein, we offered a resource to evaluate the relationships across genome, transcriptome, proteome, cell-surface proteome, and drug responses in PCa patients-derived primary cell samples. Additional examination of the pharmacoproteome uncovered PCa-related biomarkers and their corresponding treatment strategies.

## Results

### Establishment of the prostate cancer model repository (PCMR)

**Fig. 1:**
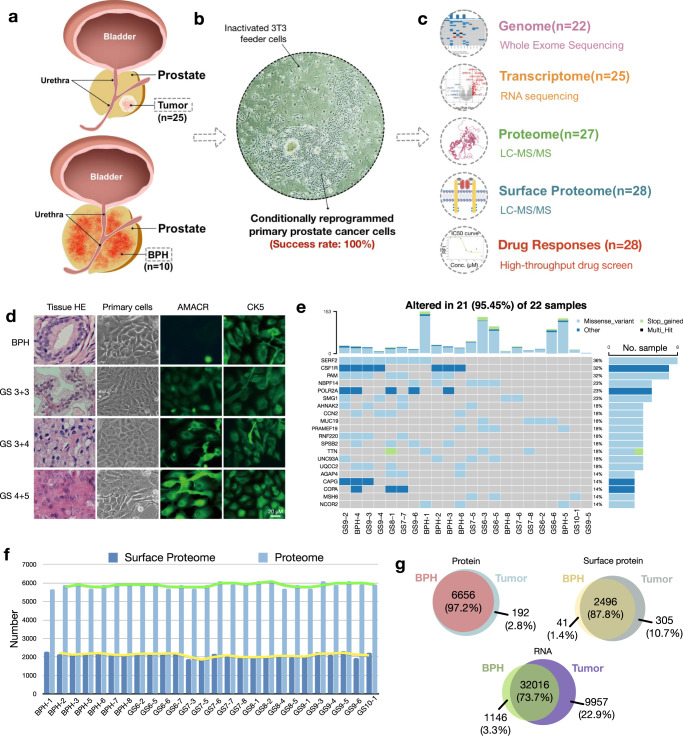
Multi-omics landscape of PCMR. **a**–**c** Workflow of multi-omics and drug sensitivity screening landscape of Prostate Cancer Model Repository (PCMR). Benign prostatic hyperplasia (BPH) or tumor primary cell samples were generated from 10 BPH and 25 Chinese prostate cancer (PCa) patients. **d** The HE staining of BPH tissue and three cases of PCa tissue, as well as the morphology and immunofluorescence staining (AMACR and CK5) of primary cells (passage 3). **e** WES for in-depth somatic variant screening. Top, mutation counts for the top 20 mutant genes in each primary cell; Right, mutation types and their frequencies. **f** Overview of the proteomics and cell-surface proteomics profile of PCMR. Bar plots show protein identification number in proteome and cell-surface proteome of paired primary cell samples (*n* = 26). **g** Overlapping genes/proteins between tumor and BPH primary cells identified by RNA-seq, proteomics and cell-surface proteomics

In this study, we developed the prostate cancer model repository (PCMR) by generating 35 primary patient-derived cells, including 10 BPH and 25 tumor samples, to characterize PCa on multi-scales (Fig. 1a–c). To generate PCa cell models, we improved the primary culture process with a new tissue digestion formula, based on previous studies (Methods).^14,15^ The tissue digesting formula elevated the primary culture success rate to 100%, supporting the long-term viability and proliferation of PCa epithelial cells. In this co-culture system, epithelial-like colonies of patient-derived conditional reprogramming cells (CRCs) developed on the irradiated murine-derived 3T3 cells (Fig. 1b, d). The culture of primary cell lines allowed the replacement of murine-derived 3T3 cells with rat tail collagen I coated dishes when primary cells proliferated stably (after 10 passages).

In total, 35 cell lines were generated from 10 BPH and 25 PCa with detailed clinical pathological information including different Gleason scores (Supplementary Table 1). In agreement with a previous report,^16^ the clinical characteristics were independent of successful CRC establishment. The feasible passages with these cell lines depended on the individual patient and were unrelated to clinical malignancy. The immunofluorescence (IF) staining and immunoblotting of the clinical PCa marker alpha-methylacyl-CoA racemase (AMACR)^17^ in BPH and PCa primary cells showed varying degrees of malignancy matched to the clinical pathology grade (Fig. 1d and Supplementary Fig. 1a). In addition, the cytokeratin 5 (CK5) exhibited epithelial characteristics^18^ in all prostate primary cells and did not differ significantly across cells (Fig. 1d and Supplementary Fig. 1a). We noticed that the androgen receptor (AR) was expressed in our early-passage primary cells (within 3 passages) but never afterwards as the cells were passaged (Supplementary Fig. 1b). By comparing the hematoxylin-eosin (HE) staining of BPH-1, GS 6-1, GS 8-1, GS 9-1 and GS 10-1, we unveiled that the malignancy degree of the primary cells matched the pathological grade of tissue origin (Supplementary Fig. 1c). These findings demonstrated that the CRC culture supported the in vitro proliferation of both benign- and malignant-derived epithelial cells and preserved the clinical traits of patients.

### Multi-omics landscape of PCMR

Multi-dimensional omics data offers an exceptional opportunity to integrate data in order to better understand disease characteristics. PCMR samples were subjected to whole-exome sequencing (WES), RNA-seq, total proteome, and surface proteome analysis after sample quality control (QC) and normalization procedures (Fig. 1c).

WES was performed on 23 samples to detect any possible somatic variants in genome, which identified 1,329 somatic variation events in primary cells (Fig. 1e and Supplementary Data 1). Our prior research demonstrated that CRCs cultivated using our technique conserved the mutational landscape of the primary tissues.^19^ Herein, we compared the tumor primary cell mutations to 1840 samples from The Cancer Genome Atlas (TCGA)^20^ and two prior studies of PCa^21,22^ from the widely used cBioPortal for Cancer Genomics (http://cbioportal.org)^23^ (Supplementary Data 1), and found 84.6% overlapping mutated genes and 15.6% unique mutations (Supplementary Fig. 2a), which likely suggests the racial differences and tumor heterogeneity. In addition, RNA sequencing (RNA-seq) was carried out in total mRNA of 25 tumor and BPH primary cells, which identified 17,558 genes with transcripts per kilobase of exon model per Million mapped reads (TPM) greater than 1 (Supplementary Data 2).

Tandem Mass Tag (TMT) labeling and mass spectrometry analysis were used for global proteomic analysis. An internal reference sample was prepared by pooling all samples at equal amounts, and then was used for the four-batch analyses. The correlation coefficients of all internal reference sample runs were greater than 0.97 (Supplementary Fig. 2b), demonstrating the good reproducibility of the proteome data. Proteome quantification of all samples exhibited a unimodal distribution and passed through the quality control procedure (Supplementary Fig. 2c). At the protein and peptide levels, proteomics analysis of all patient samples yielded a total of 7062 protein groups with a false discovery rate (FDR) of 1% (Supplementary Data 3). A total of 6848 and 6656 proteins were identified in tumors and BPH cells, respectively. On average, the proteome detected 5890 proteins per sample, with a range from 5670 in BPHs to 6086 in tumors (Fig. 1f; Supplementary Data 4).

For the surface proteome analysis, biotinylation of surface-exposed proteins was performed by using a non-permeable amine-reactive biotinylation reagent, sulfo-NHS-SS-Biotin^24,25^ with an optimized protocol (Methods). Streptavidin blotting indicated successful biotinylation (Supplementary Fig. 2d). The Spearman’s correlation coefficient of 4 samples in 2 batches was greater than 0.93 (Supplementary Fig. 2e), demonstrating the high reproducibility of the surface proteome data. The correlations of the primary samples were between 0.87 to 0.98 (Supplementary Fig. 2f). Surface proteome data from all samples also exhibit a unidirectional distribution (Supplementary Fig. 2g). Combining all the samples, we identified 2644 cell-surface proteins (proteins with GO-annotation of cell-surface/plasma membrane/cell membrane/extracellular, and proteins predicted with transmembrane topology using TMHMM, Phobius) for further analysis (Supplementary Data 4). Similar to prior studies,^26,27^ ~56% of proteins were identified as plasma membrane and membrane related proteins in total (Supplementary Fig. 2h). The number of surface proteins identified by sulfo-NHS-SS-biotin ranged from 1842 to 2330 in every single sample (Fig. 1f).

We further characterized the number of mRNA/proteins identified in BPH and tumor primary cells identified at the transcriptome, proteome, and surface proteome scales, respectively. Notably, tumor cells exhibited richer mRNA/proteins (Fig. 1g) in all scales, suggesting more active cell transduction and metabolic signaling within tumor cells. So far, our research has established a comprehensive landscape of PCa patient-derived primary cells at the genomics, transcriptomics, proteomics, and surface proteomics scales.

### PCMR captures oncogenic alterations of PCa

**Fig. 2:**
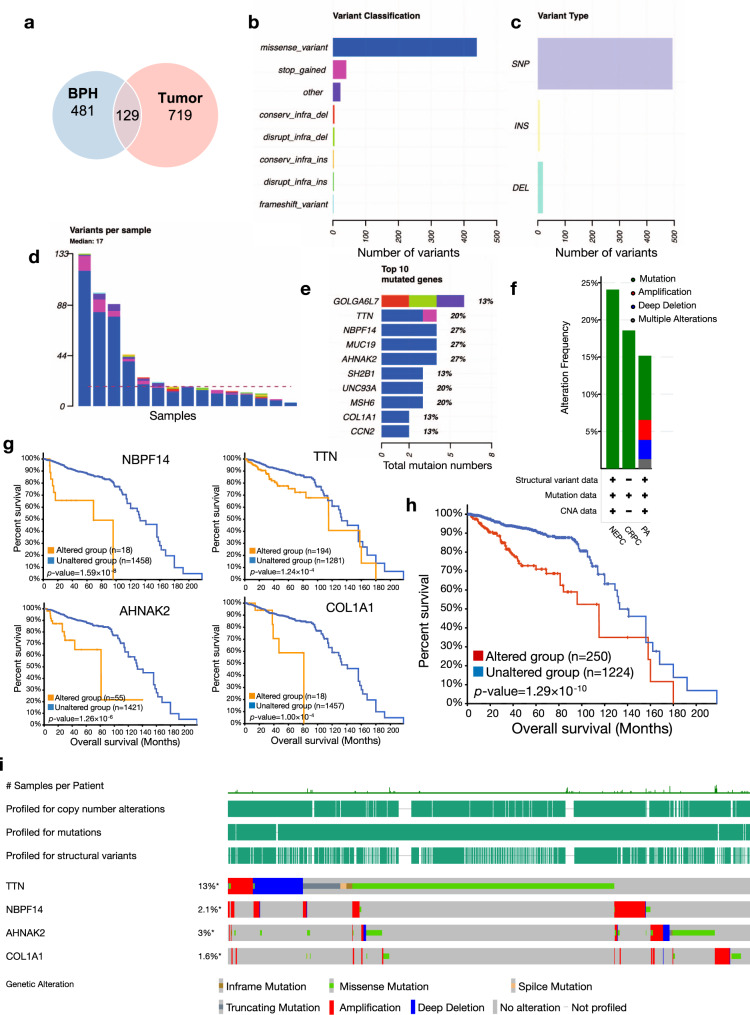
Oncogenic alterations in PCMR. **a** Overlapped mutated genes for tumor (red) and BPH (blue) primary cells identified by WES. **b** Variant classification of tumor primary cells identified by WES. **c** Variant types of tumor primary cells identified by WES. **d** The variant number of per tumor primary cell sample identified by WES. **e** Top 10 mutation genes of tumor primary cells. The fraction indicated the proportion of patients with mutation in this gene. The bar height indicated total mutations in this gene. Red, conservative in-frame deletion; green, disruptive in-frame deletion; pink, stop codon gained; blue, missense variant; purple, other variants. **f** The genomic alteration frequency of top 10 mutation genes in 7161 patients. Dark green represents mutations, blue represents deletions, red represents amplifications, and gray represents multiple alterations. Data were obtained from the cBioPortal. NEPC, prostate neuroendocrine carcinoma; CRPC, castration-resistant prostate cancer; PA, prostate adenocarcinoma. **g** The survival curve of four PCa-related genes and the combination of them (**h**) with/without gene mutations in cBioPortal. *P*-values are calculated by Log-rank test. **i** cBioPortal OncoPrint evaluate the mutations attributes of PCRCs from previous 22 studies shown in Supplementary Fig. 3a

To characterize oncogenic alterations captured by PCMR, we identified somatic mutations by WES. The results demonstrated that only a small number of mutated genes in tumors and BPH coincide (Fig. 2a), indicating different genetic backgrounds and cell proliferation mechanisms between tumor and benign samples. Variants analysis was performed to capture the genomic alterations in tumor patients (Fig. 2b–e). Comparing to 7,308 samples from 22 prior studies in cBioPortal, our study uncovered a distinctive mutational landscape of Chinese PCa primary cells that was varied from Western populations in cBioPortal (Supplementary Fig. 3a). The top 10 mutated genes in our tumor primary cell models occurred more frequently in neuroendocrine prostate cancer (NEPC) and castration-resistant prostate cancer (CRPC), which are highly malignant subtypes of PCa (Fig. 2f), partially prompting that primary cells may enriched with low-frequency malignant mutations. The top 10 high-frequency mutations (such as MUC19, NBPF14, AHNAK2, and COL1A1) detected in primary cells were rare mutations in previously reported samples except TTN (Supplementary Fig. 3a), which is known as a major mutated gene in many types of tumors including PCa. Previous research has implicated some high-frequency mutations, such as COL1A1^28^ and AHNAK2,^29^ with the invasion and metastasis in various cancers. By combining survival data in cBioPortal, we described 4 mutations (TTN, NBPF14, AHNAK2, and COL1A1) among top 10 high-frequency mutations as prognostic indicators in primary cells (Fig. 2g–i), since the other 6 genes offered less prognostic significance (Supplementary Fig. 3b). The combination of four genes provided a more significant prognostic prediction value (Log-rank test, *p*-value = 1.29 × 10^−10^, Fig. 2h). The comparison suggested that the high-frequency mutations detected in primary tumor cells may catch the malignant and rare mutations in PCa which exhibited strong prognosis value.

### In-depth multi-omics analysis of PCMR

In the present study, the differences between BPH and tumor primary cells were comprehensively characterized at four levels: genome, transcriptome, proteome and cell-surface proteome. Integrative multi-omics profiling supported the molecular understanding of the disease using an exploratory approach to improve the precision of clinical decision making. Integrated multi-omics profiling improved the precision of clinical decision making by enhancing molecular understanding of the disease. Globally, mRNA and protein abundance exhibited a moderate correlation of 0.26 (Spearman correlation coefficient) in tumor samples (Supplementary Fig. 4a) and a weaker correlation in BPHs. Several various types of tumors had exhibited weak relationships between the abundances of certain RNA transcripts and the corresponding protein.^30,31^ We validated the presence of modest transcriptome-proteome correlations in PCa primary cells. Pathway enrichment analysis of genes with relatively positive or even negative mRNA-protein correlations demonstrated that genes with strong correlations were predominantly enrolled in biosynthesis and metabolism processes, while genes with negative correlations were involved in transcription-related processes using Reactome database (Supplementary Fig. 4b). The results suggested that protein post-translational modification substantially shaped its abundance and biological process.

**Fig. 3:**
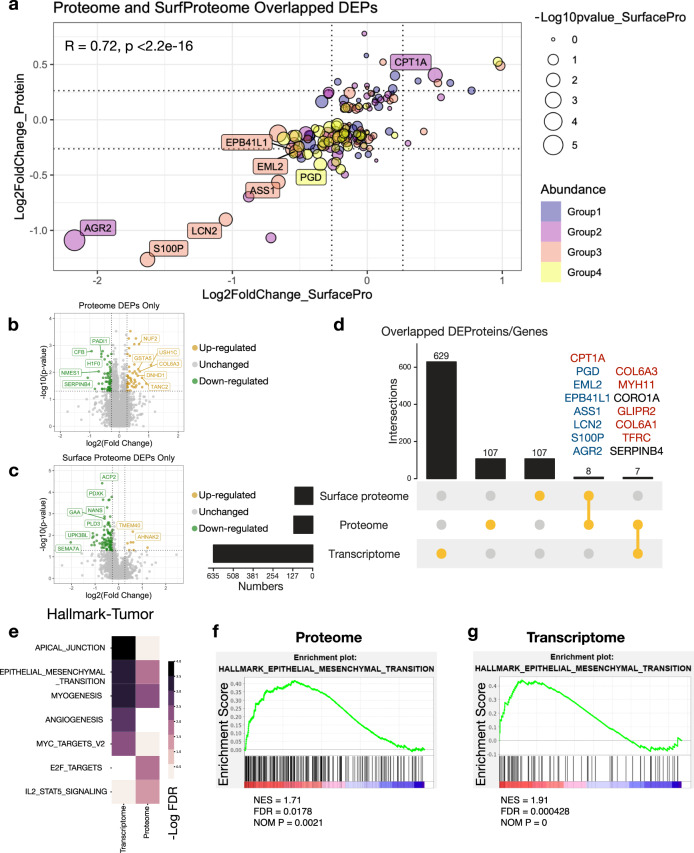
Integrative multi-omics analysis of PCMR. **a** Scatterplot of 155 matched proteins and surface proteins in PCa versus BPH-derived primary cell samples. 1770 proteins were removed due to nonsignificant *p*-values in proteome. The color represents the position of the protein in 4 equal parts of protein abundance, where yellow indicates the most abundant. The size of the circle indicates the −log10 (*p*-value) in proteome. P-value and Pearman’s correlation between protein and surface protein abundance is shown in the upper left corner of the figure. **b**, **c** Volcano plot of the differential expression proteins solely identified in proteome data (**b**) or surface proteome data (**c**). **d** Number of overlapped DEG/Ps between protein, mRNA, and cell-surface protein. Bar plot on the left indicate the total number of associated genes in each type. Bar plot on the top show the number of genes in the singleton or intersection groups as indicated by the dots below. Genes are filtered by *p*-value < 0.05 and fold change (protein) >1.2 or <0.83, fold change (surface protein) >1.2 or <0.83 and fold change (RNA-seq) >1.5 or <0.67. **e** GSEA pathway enrichment analyses of the RNA-seq and proteomic data using hallmark database revealed pathways that are significantly altered (FDR < 0.05) in tumors. **f**, **g** GSEA (Hallmark gene sets) enrichment of EMT pathway at mRNA or protein scale in tumor samples

We next performed differentially expressed gene analysis on proteome (Supplementary Fig. 4c), surface proteome (Supplementary Fig. 4d), and transcriptome scales (Supplementary Fig. 4e), in which, totally 122 proteins (Wilcoxon rank-sum test, *p* < 0.05, fold change >1.2), 115 surface proteins (Wilcoxon rank-sum test, *p* < 0.05, fold change >1.2) and 637 genes (Wald test, *p* < 0.05, fold change >1.5) were significantly changed between BPH and tumor cells, respectively. Transcriptomic data showed the most dynamic range, followed by surface proteomic data and finally proteomic data. Proteins or genes were considered significantly altered (DEP/Gs) if their fold change and p-value met the certain threshold. To compare proteome and surface proteome data, we investigated 155 DEPs that were noteworthy in proteome and surface proteome data and excluded 1770 proteins with a nonsignificant *p*-value in proteome data. The fold change of overlapped DEPs in surface protein and protein was well correlated (Pearman’s rho = 0.72, *p* < 2.2 × 10^−16^; Fig. 3a), and the cell-surface protein abundances performed a greater fold change dynamic range than the total protein abundances. The majority of DEPs were observed exclusively in whole cell lysate (114 proteins, Fig. 3b) or on the cell membrane (107 proteins, Fig. 3c), showing a distinguishable surface protein scenario. Notably, eight DEPs (CPT1A, PGD, EML2, EPB41L1, ASS1, LCN2, S100P, and AGR2) were significantly altered in both the proteome and surface proteome data (Fig. 3d).

Gene set enrichment analysis (GSEA) according to Hallmark and KEGG database demonstrated that highly expressed mRNA/proteins in tumor were highly enriched in epithelial-mesenchymal transition (EMT), myogenesis, glycan biosynthesis and extracellular matrix (ECM) receptor interaction pathways (Fig. 3e and Supplementary Fig. 4g), whereas mRNA/proteins enriched in BPHs were enrolled in inflammatory, oxidative phosphorylation and amino acid metabolism process (Supplementary Fig. 4f, h). Notably, the EMT process was highly enriched in tumor transcriptome and proteome scales (Fig. 3f, g). The result revealed that tumor cells lost epithelial phenotype and showed a more invasive pattern compared to BPH cells. It was worth noting that some enriched pathways from transcriptome or proteome data were different, such as E2F targets, IL2-STAT5 signaling and N-glycan biosynthesis pathways were only enriched in the proteome, while angiogenesis and MYC target genes are more pronounced in transcriptome enrichment (Fig. 3e and Supplementary Fig. 4f–h). These data further suggested that RNA and protein level analysis offered complementary molecular characteristics. Therefore, combining the transcriptomic and proteomic information is essential to deeply understand the cell phenotypes and disease features on multi-scale.

### Downregulation of AGR2 is a biomarker for PCMR aggressiveness

**Fig. 4:**
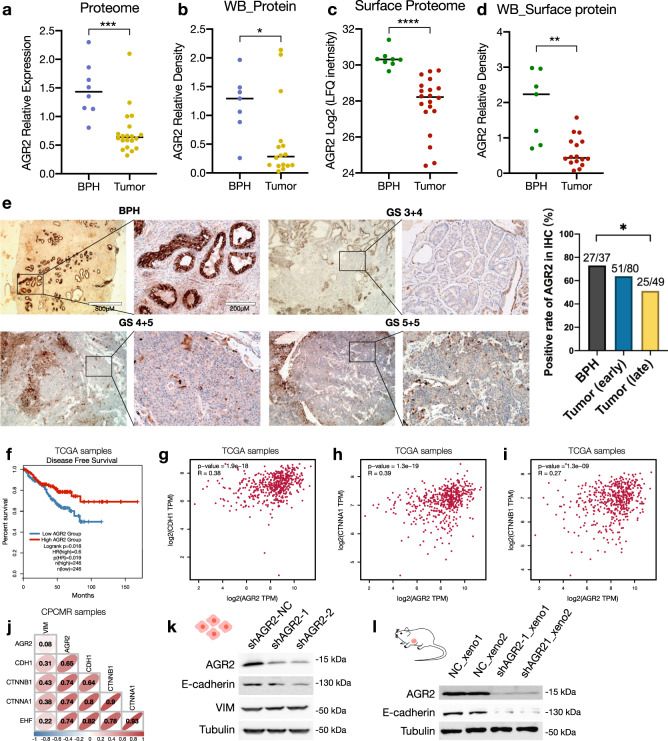
AGR2-downregulation was an invasive biomarker in PCMR. **a**–**d** The AGR2 expression of BPH-versus tumor-derived primary cell in whole cell (**a**, **b**) and surface protein (**c**, **d**) lysates was evaluated employing LC-MS/MS (**a**, **c**) and western blotting (WB, **b**, **d**). The protein blot bands of WB were quantified utilizing gray scanning (ImageJ). *p < 0.05, **p < 0.01, ***p < 0.001, ****p < 0.0001 by Wilcoxon rank-sum test. **e** IHC staining of AGR2 in different pathological grades (*n* = 138). The representative AGR2 IHC results (left) performed on Gleason score (GS) 3 + 4, GS 4 + 5, GS 5 + 5 and BPH. The positive rate of AGR2 was analyzed among BPHs (*n* = 37), early stage of tumors (GS 6–7, *n* = 80) and late stage of tumors (GS 8–10, *n* = 49) (right). *p < 0.05 by Fisher’s Exact test. Scale bar, 200 μm. **f** Kaplan–Meier plots for disease-free survival (DFS) of TCGA-PRAD patients grouped by the median of AGR2 levels. Statistics calculated by Log-rank test. **g**–**i** Scatter plots showing the Spearman’s correlation between AGR2 and three epithelium-related genes (CDH1, CTNNA1, CTNNB1) in TCGA PRAD (*n* = 494) database. The Spearman’s correlation was analyzed by GEPIA. **j** Spearman’s Correlation analysis between AGR2 and EMT-related genes (CDH1, CTNNA1, CTNNB1, EHF, VIM) in PCMR. **k**, **l** WB analysis of E-cadherin, Vimentin expression levels in NC/AGR2-KD PC3 cells (**k**) and xenograft tissues (**l**)

Differential expression analysis in multi-omics showed that AGR2 was significantly downregulated in tumor- versus BPH-derived primary cell proteome (Wilcoxon rank-sum test, 0.49-fold, *p* = 0.0002, Fig. 4a) and cell-surface proteome (Wilcoxon rank-sum test, 0.17-fold, *p* < 0.0001, Fig. 4c), and AGR2 was also comparatively lower in tumors at mRNA level (Wilcoxon rank-sum test, 0.52-fold, *p* = 0.12, Supplementary Fig. 5a). AGR2 is a member of the protein disulfide isomerase (PDI) family, which was reported to regulate protein homeostasis regulation and maintain epithelial phenotype, indicating an undeniable role in cancer progression.^32,33^ Immunoblotting analysis (Supplementary Fig. 5b) validated that the abundance of AGR2 were remarkably downregulated in tumor- versus BPH-derived primary cell lysates and membrane proteins (Fig. 4b, d) and were significantly related to the clinical malignancy of patients (Supplementary Fig. 5c). AGR2 immunohistochemical (IHC) staining was performed in an independent cohort of 37 BPHs and 129 PCa clinical samples, the positive rate of AGR2 decreased as the tumor stage progressed (Fisher’s exact test, *p* = 0.0471, Fig. 4e), indicating that the diminished protein expression levels of AGR2 were correlated with the malignancy of tumor. In TCGA datasets, low AGR2 expression levels were significantly related to poor disease-free survival (DFS) in PCa (Fig. 4f). Moreover, gene correlation analysis in TCGA datasets indicated that the mRNA expression levels of AGR2 were significantly positively correlated with epithelial markers (E-cadherin, α-catenin and β-catenin, Fig. 4g–i) but was not correlated with the vimentin and N-cadherin expression (Supplementary Fig. 5d-e). AGR2 mRNA expression levels and survival analysis were performed by GEPIA^34^ (http://gepia.cancer-pku.cn). A similar correlation trend was also found in our RNA-seq data (Fig. 4j).

To validate the correlation between AGR2 and E-cadherin, we further constructed AGR2-knockdown (AGR2-KD) PC3 cell line and corresponding PC3 xenograft nude mice models. E-cadherin expression was significantly reduced in AGR2-KD PC3 cell lines (Fig. 4k) and PC3 xenograft models (Fig. 4l). These findings partially explained that the patients with lower AGR2 expression displayed shorter DFS and trended to develop malignant phenotype and cancer metastasis. Overall, restrained AGR2 may associate with the loss of tumor epithelial properties, resulting in diminished tumor cell adhesion and performing a malignant phenotype with metastasis-associated traits. Interestingly, the reduced AGR2 expression levels were not contributed to poor prognosis in many other cancers in TCGA datasets, suggesting that AGR2 may play a unique role in PCa (Supplementary Fig. 6).

### Diversified pharmaceutical responses in PCMR models

**Fig. 5:**
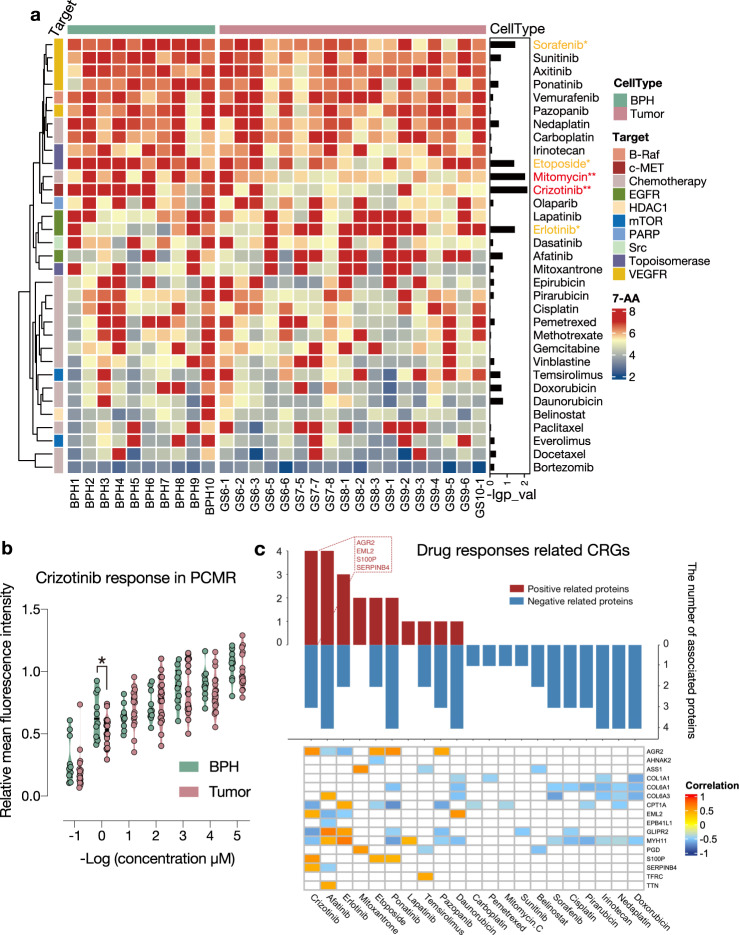
Diversified drug responses in PCMR models. **a** Heatmap showing drug responses in 28 PCMR models (left) and bar plot showing the p-value of drug response between BPH and tumor primary cell models (right). Pink represents tumor and green represents BPH. Drug–response value is presented as the 7-Activity Area (7-AA). **b** The mean fluorescence intensity of BPH (*n* = 10) and tumor (*n* = 18) primary cell viability as measured by CellTiter Glo after 72 h of crizotinib treatment. The fluorescence intensities are normalized to the DMSO treatment of each cell line. **c** Pharmacoproteomic interaction of CRGs in PCMR. Bar plot showing the number of proteins significantly associated with drugs (*p*-value <0.05), Red represents for positively related proteins, blue represents for negatively related proteins. Heatmap showing the specific correlation values of protein-drug pairs and nonsignificant correlations (*p*-value > 0.05) have been hidden in white. *P*-values are calculated by unpaired two-sided Student’s *t* test. *p < 0.05, **p < 0.01

We next performed an in vitro drug screening to characterize diverse pharmaceutical perturbations in PCMR (Fig. 5a). We assembled 33 anticancer drugs authorized by the FDA, comprising 15 chemotherapeutic and 18 multi-targeted drugs against nine cellular pathways (Supplementary Data 5). Twenty-nine PCMR models were screened (Method, Supplementary Fig. 7a) against this drug panel with 7 concentrations for each drug, yielding >6,699 measurements of cell-drug associations. Half-maximal inhibitory concentration (IC_50_) and activity area (AA) of each cell-drug interaction were determined to reflect pharmaceutical responses in PCMR models^11,16^ (Supplementary Data 6), and they were shown to be highly correlated (Supplementary Fig. 7b). In general, drugs with the same target had a tendency to be clustered together, which suggested the credibility of drug screening results (Fig. 5a). Interestingly, we identified five drugs (sorafenib, etoposide, mitomycin C, crizotinib, erlotinib) that showed selective responses among BPHs and tumors (t.test, *p* < 0.05, Fig. 5a). The most effective selective drug was crizotinib, a kinase inhibitor of the receptor tyrosine kinase anaplastic lymphoma kinase (ALK) and the c-Met/hepatocyte growth factor receptor (HGFR) (Fig. 5a). The dose-response plot for crizotinib in all cell lines exhibited a significant sensitivity at 1 μM (Fig. 5b), indicating that tumor primary cells are more susceptible to crizotinib-induced inhibition. Although the molecular mechanisms of these drugs for PCa were unclear, our dataset contained pharmacological candidates and their repurposing for PCa therapy. Our observations revealed that the high-throughput drug screening in PCMR elicited a variety of drug responses and offered prospects for pharmacoproteomic analysis in PCa.

### Pharmacoproteomic analysis in PCMR

**Fig. 6:**
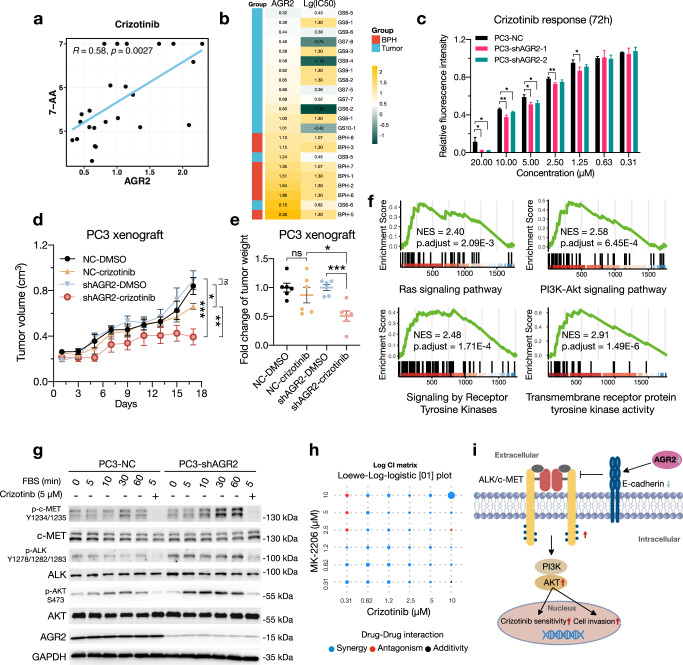
Crizotinib sensitivity in PCa was negatively affected by AGR2. **a** Spearman correlation analysis between crizotinib (7-AA values) and normalized AGR2 protein intensities in PCMR. **b** The protein expression levels of AGR2 and log10 (IC_50_) of crizotinib in PCMR. **c** Viability (relative fluorescence intensity) of NC/AGR2-KD PC3 cells treated with crizotinib for 72 h as measured by CellTiter Glo. The data are presented as mean ± SD. The fluorescence intensities are standardized to the DMSO treatment of each cell line. **d** The tumor volume of NC/shAGR2-2 PC3 cells after subcutaneous inoculation into nude mice and treated with 25 mg/kg crizotinib every two days. Tumor volume was measured once every two days for 18 days. The data are presented as mean ± SEM, *n* = 6 mice/group. **e** Normalized fold change of tumor weight. Tumor weight data were normalized to the DMSO treatment of each group. The data are presented as mean ± SEM, *n* = 6 mice/group. **f** GSEA enrichment plots of Ras, PI3K-Akt, RTKs and transmembrane RTK activity pathways. **g** WB analysis of p-AKT (S473), p-ALK (Y1278/1282/1283) and p-c-MET (Y1234/1235). PC3-NC/shAGR2 cells were treated with 10% FBS prior to crizotinib (5 μM) treatment or left untreated. **h** Log CI values show for PC3-shAGR2-2 cell line treated with the drug combination of MK-2206 and crizotinib for 72 h. The higher the size of the circle is, the higher the CI power. **I** Proposed work diagram. *P*-values are calculated by unpaired two-sided Student’s *t* test. *p < 0.05, **p < 0.01, ***p < 0.001

Pharmacoproteomic analysis was an unbiased approach for identifying possible protein markers in response to drugs. Overall, we identified 14,372 significant protein-drug interactions (Supplementary Fig. 7c, Supplementary Data 6). To further confirm functional proteins associated with drug efficacy and response, we identified 19 genes as cancer-related genes (CRGs) in PCa, whereas 15 were overlapped DEP/Gs (Fig. 3c), and four (TTN, NBPF14, AHNAK2, COL1A1) were the top mutated genes in PCMR (Fig. 2g, I). We identified 89 CRG-drug pairs with significant interactions, in which the drug responses of crizotinib and afatinib were associated with the highest CRG protein expression levels (Methods, Fig. 5c and Supplementary Data 6). Specifically, seven CRGs were significantly related to crizotinib response (Fig. 6a and Supplementary Fig. 8), of which, four proteins were positively correlated (AGR2, EML2, S100P, SERPINB4) and three proteins were negatively correlated (CPT1A, GLIPR2, MYH11). Unexplored protein-drug associations that could aid clinical design and hasten precision medicine in PCa need proper confirmation.

Collectively, PCMR offered a protein-drug association resource that can be investigated as possible predictors of pharmaceutical responses. We detected affluent protein markers of drugs, including associations that cannot be explained by our existing understanding. With adequate validation studies, these potential indicators may contribute to patient classification and clinical response prediction.

### AGR2 deficiency enhanced the crizotinib inhibition in PCa

Since crizotinib performed best drug selectivity for PCa in our screening (Fig. 5a), and AGR2 was the most significant DEP (Fig. 3a) between PCa- and BPH-derived primary cells, the significantly positive correlation (Spearman R = 0.58, *p* = 0.0027; Fig. 6a, b) between crizotinib and AGR2 was of considerable interest. We wondered if AGR2 was liable for fine-tuning the inhibition activity of crizotinib. In vitro, crizotinib showed a more pronounced inhibition effect on PC3 with AGR2-KD in a dose-depended manner (Fig. 6c and Supplementary Fig. 9a). The growth rate of PC3 cells with AGR2-KD was first drastically inhibited but resumed after a week, which might be the result of parallel signaling activation. (Supplementary Fig. 9b, c). In vivo, xenografts with AGR2-KD performed elevated tumor suppression compared to PC3-NC in response to crizotinib (Fig. 6d, e and Supplementary Fig. 9d, e) without showing signs of severe toxicity (Supplementary Fig. 9f), suggesting that AGR2 loss exacerbated crizotinib-induced inhibition and may serve as a biomarker for crizotinib responses in vivo.

To investigate how AGR2 orchestrated the crizotinib sensitivity, we performed GSEA pathway enrichment analyses of RNA-seq and proteomics data on three BPHs (BPH1, BPH2, BPH5) and three tumor (GS6-2, GS7-8, GS9-4) primary cells that displayed the most noticeably varied crizotinib susceptibility and AGR2 expression levels (Fig. 6b). Both principal component analyses (PCAs) of RNA-seq and proteomics data were able to distinguish between these tumors and BPHs, with the transcriptomics analysis providing the optimal separation (Supplementary Fig. 10a, b). We next performed differentially expressed gene analysis which identified 1747 genes (Wald test, *p* < 0.05, fold change > 2) and 308 proteins (t.test, *p* < 0.05, fold change > 1.2) that significantly altered between BPHs and tumor samples. GSEA indicated that tumors significantly activated pathways such as Ras, PI3K-Akt, signaling by receptor tyrosine kinases (RTKs) and transmembrane receptor protein tyrosine kinase activity (Fig. 6f and Supplementary Fig. 10c–e). These results partially prompted that the restrained AGR2 expression activated the RTK signaling, which contributed to enhanced the inhibition performance of crizotinib.

Since crizotinib is a well-known dual inhibitor of the c-Met and ALK,^35^ we utilized western blot to examine the activation changes of p-ALK (Y1278/1282/1283), p-c-MET (Y1234/1235) and p-AKT (S473) in AGR2-KD cells. The phosphorylation signaling in PC3-shAGR2 cells was more activated by FBS stimulating which was greatly inhibited by crizotinib (5 μM) treated for 5 min (Fig. 6g). We further evaluated the synergy of crizotinib with MK-2206, a highly selective AKT1/2/3 inhibitor which strikingly reduced AKT signaling.^36^ We employed the Loewe additivity model as the combination index (CI) model and the Log-logistic[01] with three parameters as the drug–response curve model using the app SiCoDEA.^37^ Combinations of MK-2206 (between 0.31 and 1.2 M) with crizotinib (between 0.62 and 10 M) in the PC3-shAGR2-2 cell line demonstrated a substantial synergy (Fig. 6h and Supplementary Fig. 10f, g).

These results revealed that AGR2 boosted ALK/c-MET signaling, which enhanced the performance of crizotinib inhibition efficacy. Notably, diminished AGR2 downregulated E-cadherin (Fig. 4k, l), and a prior study has indicated that E-cadherin downregulation may aid in the ligand-dependent activation of RTK in tumors.^38,39^ Together, these data unveiled that the low expression of AGR2 diminished E-cadherin expression to activate the ALK/c-MET signaling which was closely related with the enhanced response to crizotinib (Fig. 6i) in PCa.

## Discussion

We here reported a comprehensive multi-omics analysis of 35 Chinese PCa primary cell lines, quantifying more than 1800 mutations, 17,000 transcripts, 7000 proteins, 2000 cell-surface proteins as well as responses to 33 FDA-approved drugs. Using integrated multi-omics analysis, we discovered that disease nature in PCMR patients is correlated with genomic abnormalities, transcriptome expressions, and protein/surface protein abundance, which could be applied to predict drug sensitivity relevant to precision medicine.

Cell membrane surface proteins are the first line of defense against the environment and drugs. In-depth screening of cell surfaceome remains challenging due to the characteristics of low abundance and high hydrophobicity. We knew very little about the correlation between membrane proteins and drug sensitivity. Our study utilized an optimized protocol^25^ allowing reduced number of starting cells (10^5^-10^6^ cells per experiment) to enrich and identify membrane proteins and assessed the potential interaction between membrane proteins and drugs based on primary cell lines. Generally, the coverage of proteome is not as deep as transcriptome yet owing to technical limitation. Since the number of proteins identified can be influenced by many factors such as cell type, sample preparation, LC gradient and type of MS instrument. Further efforts in optimizing sample preparation and LC-MS/MS parameters will benefit the improvement of the depth and internal concordance of molecular analysis.

Tumor heterogeneity in PCa elicited a complex molecular classification for patients.^40^ Compared with PCa tissue studies, top mutant genes, such as TP53 and SPOP, were not mutated in PCMR. This may be a result of the dominant population screened during CRC cultivation.^16^ Recent data revealed that metastasis was typically spread by a tiny group of 10–150 cells in the tumor interior.^41,42^ Notably, most of the drug-targeted mutations occurred in the very early stages, as these variants were present in the majority of tumor cells^43^ and were easily detected by whole-tissue sequencing. However, cells in tumor became heterogeneous with tumor evolving, while a small subset of cancer cells acquired new mutations in advanced stages, contributing to poor prognosis.^44^

Both BPH and PCa could contribute to cell extensive proliferation, finding out why tumor cells performed a more aggressive behavior than BPH cells was indispensable for understanding tumor progression. Our study showed that AGR2 protein expression levels in tumors were significantly diminished compared to BPHs, which implied that AGR2 considerably orchestrated the aggressive phenotypic differences between malignancies and benign hyperplasia, such as migration and metastasis. AGR2 plays a crucial role in embryonic development and tissue regeneration^45^ and has been linked with the initiation and development of several cancers, including breast,^46^ lung,^47^ ovarian,^48^ pancreatic,^49^ and prostate^32,33^ cancers. Most speculation were over AGR2’s pro-proliferative function, even using it as a tumor biomarker.^46–49^ This was primarily supported by the elevated AGR2 expression in tumors, and overexpression of AGR2 would promote cell growth. However, these findings cannot account for the fact that lower AGR2 expression was strongly related with a poor prognosis in PCa (Fig. 4f). Especially, metastasis is typically spread by a tiny population inside the tumor,^41,42^ leading to a poor prognosis. We proved a substantial positive correlation between AGR2 and E-cadherin in transcriptome and WB, further indicating a potential association between AGR2 and EMT process and supporting earlier discoveries that AGR2 abrogation greatly reduced cellular attachment^50^ and showed significantly elevated metastases lesions.^47^ In addition, a prior limited study employed an IHC analysis^33^ demonstrated the CD10^high^AGR2^low^ subtype was more prevalent in high-grade initial tumors and speculated that AGR2 performed a protective function in primary tumors but may contribute to the distant dissemination of tumor cells. Taken together, AGR2 appears to have a promoting role in multiple primary tumors, but we confirmed that its downregulation was crucial for regulating distant dissemination of PCa cells.

PCMR provided primary cell models to faithfully assess the efficacy of drugs and took individual differences into account. Herein, we underlined the selectivity of crizotinib between BPHs and tumors while unveiled possible explanations based on multi-omics scale. Agents targeting AR signaling remained the cornerstone for PCa therapy, however, stringent AR disruption elevated c-MET expression, which contributed to PCa progression.^51^ Crizotinib performed an anti-proliferative activity for advanced PCa especially when paired with androgen ablation therapy, according to preclinical studies performed in cell lines, organoids and xenograft models.^52–54^ Despite a phase I study discovered that 74% steady-state crizotinib diminished when gave along with enzalutamide,^55^ this pathway remained crucial in PCa, and trials combining novel AR and c-MET inhibitors with fewer probable pharmacokinetic interactions were under investigation. Our multi-omics investigation revealed that crizotinib selectively inhibits PCa cells, particularly AGR2-deficient PCa cells. These results improved our knowledge of crizotinib-induced inhibition and supported future therapeutic combinations. More drugs and biomarkers could be achievable with more validation, hastening the progress of PCa therapeutic development.

However, it should be noted that AR is only expressed on prostate luminal epithelial cells but the conditional reprogramming (CR) -based PCMR model are stem-like transit-amplifying epithelial cells^56–58^ which cannot sustain AR expression (Fig. 1d, Supplementary Fig. 1b). The findings were highly consistent with the established PCa primary cells.^59^ However, the use of AR-negative primary cell lines to explore androgen-derived treatments may be limited. Moreover, the CR-based 2D cell model that does not possess a tumor microenvironment and therefore has limited representation with in vivo drug responses. Organoids and PDXs have superiority in maintaining cancer tissue structure and complex microenvironment,^54^ but 2D cells were comparatively simple to expand in large quantities, allowing for pharmacoproteomic analysis. Pharmacoproteomic analysis indicated the possible proteins and related pathways affected by drugs on proteome scale, avoiding the epigenetic effects and post-translational modifications compared to pharmacogenomic. Matching the pharmaceutical responses with protein abundances to provide insights into cancer biology and to promote the clinical decision making. These putative biomarkers may facilitate patient classification and contribute to the explanation of variability in patient clinical response if they are validated through the necessary investigations.

In conclusion, our study established a paradigm for enhancing drug discovery by employing multi-omics in cell platforms. We constructed a knowledge base on protein-drug interactions in PCa, which, if adequately confirmed, might aid clinical decision making and speed up the development of precision medicine. We hope to conduct biological functions and clinical trials of numerous candidate drug-protein associations, and to improve the accuracy with the larger datasets in the future.

## Materials and methods

### Patient sample collection

Prostate tissue specimens were collected from patients who underwent prostate biopsy or radical prostatectomy from Huashan Hospital, Fudan University, consisting of 10 cases of BPHs (number BPH-1 to BPH-10), 7 cases with Gleason score of 6 (number GS 6-1 to GS 6–7), and 6 cases with Gleason score of 7 (number GS 7-3 to GS 7-8). Five cases with Gleason score of 8 (numbers GS 8-1 to GS 8-5), six cases with Gleason score of 9 (numbers GS 9-1 to GS 9-6) and one case with Gleason score of 10 (numbers GS 10-1). Among them, the sample of GS 6-3 was obtained from PCa with seminal vesicle invasion, the samples of GS 8-1 and GS 9-3 were obtained from patients who were sensitive to androgen deprivation therapy. The samples of GS 8-2 and GS 10-1 were obtained from patients who were insensitive to androgen deprivation treatment (CRPC).

Pathological examination diagnosed all involved patients with PCa or BPH (Supplementary Table 1). Ethics Committee at Huashan (Shanghai, China; approval number: KY2011-009) and Zhongshan Hospital (Shanghai, China; approval no. B2019-247R) approved the study procedure. Before collecting samples from patients, informed consent was acquired. The experimental methods were executed in conformity with the Huashan and Zhongshan Hospitals’ authorized Ethics Committees. Immunohistochemistry (IHC) was performed on formalin-fixed, paraffin-embedded pathological specimens from the repository. The electronic patient record system at Huashan and Zhongshan hospitals was mined for patient clinical data.

### Cell model generation and immunofluorescence staining

To establish cell models from clinical specimens, we followed the CR cell culture medium.^14^ Specifically, the primary culture medium was prepared by combining DMEM (Gibco, C11995500BT) and F-12 nutrient (Gibco, 11765-054) as a fix ratio of 3:1 and supplementing it with 5 µg/mL insulin (Sigma-Aldrich, I-5500), 0.125 ng/mL human epidermal growth factor (Gibco, PHG0313), 25 ng/mL hydrocortisone (Sigma-Aldrich, H-0888) and 10% fetal bovine serum (Gibco, 2175442P). The medium was filtered through a 0.2-µm sterile filter and kept at 4 °C after adding 10 µM ROCK (Rho-associated coiled-coil-containing kinase) inhibitor Y-27632 (DC Chemicals, DC1028) up to 2 weeks. Tumor-derived cell pellets were transferred to a culture dish containing feeder cells at a density of 1 × 10^4^ cells/cm^2^ in primary media. The formation of obvious tumor colonies was seen 24–72 h after seeding.

Briefly, fresh tissue was obtained from Chinese patients by surgical excision or biopsy (Supplementary Table 1). Tissues with necrosis were discarded. The remainder was sliced into 2 to 3 mm fragments with scissors and digested with 0.15% Collagenase Type I (Sigma-Aldrich, C0130), 0.1% Dispase (Gibco, 17105-041), and 0.04% Hyaluronidase (Sigma-Aldrich, H3506) in primary medium for 30–180 min at 37 °C. The cell suspension was then filtered using a 70 μm cell strainer and centrifuged at 1000 revolutions per minute for 5 minutes. The cancer cells were resuspended in primary medium, transferred to feeder cell-coated plates, and then cultured in a humidified incubator with 37 °C and 5% CO_2_. Frequent microscopy was utilized to observe the expansion and proliferation of epithelial cell clones. Picking out epithelial cell clones were picked out to avoid fibroblast contamination. Once cells reached 80% to 90% confluency, epithelial cells were enzymatically detached with 0.25% trypsin-EDTA (Gibco, 25200114) and passaged at a ratio of 1:3. Fresh primary medium was changed every 2 to 3 days. Most primary cells could proliferate on dishes coated with rat tail collagen I (Corning, 354236) after 10 passages, but cannot proliferate normally as epithelial phenotype without collagen. Few primary cells lost their epithelial features after 20 passages, but the majority of initial cells could undergo more than 30 passages. To close the patient’s tumor characteristics as much as possible, we manage all of our sequencing within 2–5 passages to minimize the impact of mutations and status changes caused by in vitro culture. Primary cell lines were publicly available.

To validate the features of primary cells, immunofluorescence labeling was performed. After primary cell digestion, inoculate a 24-well culture plate at a density of 2000 per well. Following 24 h, the slides were washed three times with PBS and fixed at room temperature for 15 minutes with 4% paraformaldehyde. The slides were then washed with PBS three times. The slides were perforated for 20 minutes at room temperature with 0.5% Triton X, washed three times with PBS, and then incubated for 30 minutes with 3% BSA. Dropwise add diluted AMACR (Abcam, ab246927, 1:100) or CK5 (Abcam, ab52635, 1:100) and incubate at 4 °C for 12 h. The slides were washed three times with fluorescent secondary antibody coupled with primary antibody, incubated for 1 h at 37 °C in a humid box, and then rinsed three times with PBST. Add DAPI dropwise to cover the plate in the dark for 5 minutes and acquire images.

### Subcutaneous xenograft

PC3 and its knockdown cell lines were grown in RPMI 1640 (Gibco) medium with 10% FBS, 1% penicillin/streptomycin and GlutaMAX (Gibco), in a 37 °C incubator with 5% CO_2_. The PC3-NC and PC3-shAGR2-2 cells were amplified and resuspended in PBS. A total of 2 × 10^6^ PC3-NC and PC3-shAGR2-2 cells mixed with Matrigel (354234, Corning, Corning, NY) were injected subcutaneously into the flank of male nude mice aged six weeks. In the 7th day after injection cells, the diameter of the tumor reached around 0.5 cm. Crizotinib was administered at 25 mg/kg once every two days by intraperitoneal injection. The tumor size was measured with a caliper during the entirety of the experiment. The volume of the tumor was determined using the formula: v = 0.5 × a × b^2^ (v, the tumor volume; a, the major diameter of the tumor; b, the minor diameter of the tumor). The animals were housed in a facility free of particular pathogens (12 h light/dark cycle, 21–23 °C temperature, and 30–70% relative humidity). All animal experiments were conducted in compliance with the Animal Care & Use Committees of Department of Laboratory Animal Science, Fudan University (approval number: 2022JS Huashan-160).

### Whole-exome sequencing

MagPure Tissue & Blood DNA LQ Kit was used to extract genomic DNA (D6312-02). Biorupter (Diagenode, Belgium) was used to generate 150–200 bp fragments from 200 ng genomic DNA from each individual. DNA fragment ends were repaired, and an Illumina Adaptor was attached (Fast Library Prep Kit, iGeneTech, Beijing, China). Following the preparation of the sequencing library, the whole exons were collected with AIExome Enrichment Kit V2 (iGeneTech, Beijing, China) and sequenced on the Illumina NovaSeq 5000 platform (Illumina, San Diego, CA) with 150base paired-end reads. FastQC (version 0.11.9) was used for quality assurance. Linkers and low-quality readings were eliminated using Trimmomatic (version 0.39) software.^60^ We eliminated adapters and leading N bases that were of lower-than-grade 3 quality. We examined the scans with a sliding window that was four bases wide, eliminating reads when the average quality per base fell below 15 and discarding those that were less than 36 bases long. The clean data were aligned to human reference genome (UCSC_hg38) and mouse genome (mm10) by software BWA MEM (Version: 0.7.17-r1188).^61^ Use the software Samtools [Version: 1.9 (using htslib 1.9)]^62^ to convert sam format files to bam format files, sort and build indexes. R package XenofilteR (v1.6)^63^ was then used to remove the murine genes in the bam file. Duplicate reads were removed using Picard (v2.23.8-0) software. Base quality recorrection (BQSR) was performed using GATK software (v4.1.9.0),^64^ using Mutect2 to call SNP and indel following best practice. Results are annotated using ANNOVAR.^65^ Downstream analysis uses the R package maftools (v2.6.05). Because of the absence of matched normal tissues in the available PCa cell models, we employed the frequently used workflow to call probable somatic mutations in cancer cell model analysis.^16,66^ The variations were compared to recognized databases of germline variation, such as dbSNP146, 1000 Genomes, and GnomAD. If the variant was present in the COSMIC database, it would be maintained. The remaining variants were considered to be potential somatic mutations.

### RNA sequencing

Trizol (Invitrogen) was used to extract total RNA in accordance with the manufacturer’s instructions. Illumina Novaseq 5000 was used to perform paired-end 150 bp sequencing on all 25 cell models, per the manufacturer’s instructions. Before to applying any data filtering criteria, the RNA-Seq data quality was evaluated with the FastQC (version 0.11.7) program. Using HISAT2 (v2.2.0),^67^ reads were mapped to the human reference genome (GRCh38 assembly). XenofilteR (v1.6) R packages^63^ were used to screen out mouse genes after mapping. StringTie software (v2.1.2) and the genome annotation file (EMBL Homo sapiens.GRCh38.101.gtf) were utilized to assemble the mapped reads into transcripts or genes. The retained genes had a raw count greater than one, resulting in a total of 19,359 gene IDs. TPM (transcripts per million) was utilized to standardize the expression profiles (Supplementary Data 2).

The relative abundance of the transcript/gene was quantified using a metric standardized by the R package DESeq2 (v1.28.1).^68^ DESeq2 identified upregulated DEGs as those with a *p*-value <0.05 and a fold change ≥1.5, and downregulated DEGs as those with a *p*-value < 0.05 and a fold change ≤ −1.5. GSEA was conducted utilizing the Desktop Application^69^ and clusterProfiler^70^ R package.

### Proteomic analysis

#### Protein extraction

Cells were washed with PBS buffer three times and lysed in 8 M Urea in 100 mM NH_4_HCO_3_ containing protease and phosphatase inhibitors (Roche, Mannheim, Germany). Then, samples were incubated on ice for half an hour and sonicated for 4 min (2 s of sonication time at 5 s intervals). Next, the protein solution was transferred into a clean tube after centrifugation at 21,130 *g* at 4 °C for 15 min. Protein concentration was determined with a BCA protein assay kit (Beyotime Biotechnology, Shanghai, China).

#### In-solution digestion

Protein lysates were reduced by 5 mM dithiothreitol (DTT) at 56 °C for 30 min, and then incubated with 15 mM iodoacetamide (IAA) at room temperature away from light for another 30 min. After incubation, 30 mM cysteine was added to quench the alkylation reaction. Next, the protein solution was subjected to trypsin digestion with enzyme-to-substrate ratio of 1:50 at 37 °C for 16 h. Trypsin was added again at a ratio of 1:100 (w/w) for 4 h at 37 °C. Finally, those peptides were desalted by a Sep-Pak C18 column (Waters, Milford, MA, USA)

#### Tandem mass tag (TMT) labeling

10-plex TMT reagents were used to label the desalted peptides from each sample according to the manufacturer’s instructions (Thermo Fisher Scientific, San Jose, CA, USA). Internal reference sample was prepared by mixing all samples at equal amounts, and then was used in Channel 126 throughout the sample analysis. The labeling efficiency of TMT reagents was checked with an EASY-nLC 1200 system coupled to an Q Exactive HF-X mass spectrometer (Thermo Fisher Scientific, San Jose, CA, USA). After labeling efficiency confirmation (TMT modification ratio > 95% for both lysine residue and peptide N-termini), the peptides labeled by different TMT reagents were mixed with equal contribution, dried using Speed-Vac, and desalted by SepPak C18 cartridges (Waters, Milford, MA, USA). In total, the samples were labeled into four batches in the TMT 10-plex experiment.

#### HPLC fractionation for proteomic analysis

To reduce the complexity of the tryptic peptides, high-pH reversed-phase HPLC with a Waters XBridge Prep C18 column (5 μm particles, 4.6 × 250 mm) was applied to separate the TMT labeled peptides. Mobile phase A composed of 2% acetonitrile and ammonium hydroxide solution (pH = 10). Mobile phase B contained 2% mobile phase A and 98% acetonitrile. The TMT labeled peptides were dissolved in mobile phase A. After sample loading, tryptic peptides were separated with a 97 min gradient at a flow rate of 1.0 mL/min. The LC gradient started with an increase of solvent B to 5% in 2 min, 5% to 12% B for 8 min, followed by linear rise to 33% B in 57 min, 2 min to 95% B, then constantly 95% B in 13 min and another 12 min for 5% B. Finally, the peptides were incorporated into twenty fractions and dried by a Speed-Vac for further experiments.

#### LC-MS/MS analysis

LC-MS/MS analysis was performed by Q Exactive HF-X mass spectrometer (Thermo Fisher Scientific, San Jose, CA, USA) following an EASY-nLC 1200 system (Thermo Fisher Scientific, San Jose, CA, USA). A homemade reverse-phase C18 column (21 cm × 75μm column containing ReproSil-Pur 120 C18-AQ, 1.9 μm particle size, 120 Å pore size, Dr. Maisch GmbH, Germany) was used to further separate peptides. First, peptides were dissolved in mobile phase A (0.1% formic acid in 2% acetonitrile). After sample loading, peptides were eluted with a 70 minutes gradient from 6 to 30% mobile phase B (0.1% formic acid in 90% acetonitrile) in 57 minutes, 30 to 45% mobile phase B in 4 minutes at a flow rate of 300 nL/min, then 45 to 80% mobile phase B in 4 min, 5 minutes for 80% B at a flow rate of 400 nL/min. After nanoflow HPLC, precursor spectra were collected from *m/z* 350–1550 with a resolution of 60,000 at *m/z* 200, the automatic gain control (AGC) of 3e6 and maximum injection time (MIT) of 45 ms. In MS/MS acquisition, the 20 most intense ions were chosen to be fragmentized by Higher-energy Collision Dissociation (HCD) with the normalized collision energy (NCE) of 32%, then the fragment ions were detected in the Orbitrap with a resolution of 45,000 at *m/z* 200. AGC was set to 1e5, and the MIT was 30 ms. The isolation window was set to 0.8 *m/z* and dynamic exclusion duration was 30 s.

#### Proteomic database search

All MS/MS spectra were analyzed using MaxQuant software (1.6.7.0) against the Uniprot human database including 96,464 sequences (downloaded in September 2019). TMT 10-plex-based MS2 reporter ion quantification with a mass tolerance of 0.003 Da was selected. To reduce the interference of precursor co-fragmentation, the precursor intensity fraction (PIF) filter was set at 0.75. Cysteine carbamidomethylation was included as fixed modification. Methionine oxidation and protein N-term acetylation were set as variable modifications. Less than six modifications per peptide were required for each peptide. Enzyme specificity was set as trypsin/P. The maximum missed cleavages were set as two. The tolerances of first search and main search for peptides were set at 20 ppm and 4.5 ppm, respectively. The FDR cutoff for protein level was set as 0.01. For each batch of TMT labeling data, the purities of TMT labeling channels were corrected based on the kit LOT number.

#### Proteomic data analysis

##### Data normalization

Proteins from the reverse database or potential contaminant database were removed. For each sample, the reporter ion intensities from the same gene were grouped. Then the intensity was normalized by the median in each sample to calibrate sample loading differences. After calculating relative abundance as the ratio of sample abundance to internal reference sample abundance, the data were log2-transformed for further analysis.

##### Quality control and assessment of LC-MS/MS data

The density plot of the normalized intensities of the proteins quantified in each sample and dip statistic test was used to examine whether all samples passed the quality control with expected unimodal distribution. Pearson correlation of internal reference samples from different batches were used for evaluating the reproducibility of TMT labeling experiments.

##### Differential protein analysis

The Wilcoxon rank-sum test was utilized to identify proteins that varied significantly between tumor and BPH. Significant upregulated proteins were defined as those with a *p*-value <0.05 and a fold change >1.2, whereas downregulated proteins were defined as those with a *p*-value <0.05 and a fold change <1/1.2.^71^

### Surface proteomic analysis

#### Cell-surface proteome profiling by amine-reactive cell-surface biotinylation method

Cell-surface proteins were enriched by covalently coupling with primary amines via sulfo-NHS-SS-biotin.^25^ Briefly, primary cells were washed twice with ice-cold PBS and then labeled for 30 min at 4 °C with 0.25 mg/ml sulfo-NHS-SS-biotin. Subsequently, the cells were subjected to quench buffer of 100 mM glycine and then washed twice with 20 mM Tris-HCl and 150 mM NaCl (pH7.4). The cells were lysed in ice-cold radioimmunoprecipitation assay (RIPA) buffer with 0.2% (v/v) protease inhibitors (pH 8.0). Streptavidin agarose beads were then incubated with the biotinylated cell lysates for 3 h at room temperature, followed by six washes with PBS. The biotinylated proteins were eluted with PBS containing 50 mM DTT, 6 M urea, and 0.2% SDS for 30 min at 65 °C. This procedure was repeated once for complete elution. The combined proteins were digested with trypsin (1:25, w/w) via filter-aided sample preparation (FASP) method. Finally, the digest was acidified to 3% formic acid (FA) and was then dried for the following reverse-phase liquid chromatography–tandem mass spectrometry (RPLC-MS/MS) analysis.

#### RPLC-MS/MS analysis and data searching

The dried peptides were resuspended in 0.1% FA and analyzed with a Q Exactive HF mass spectrometer. Each sample was analyzed by two LC-MS/MS runs. All of the raw files were loaded onto Proteome Discoverer (versions 2.1.1.21) incorporated with mascot (versions 2.5, Matrix Science Inc.) for protein identification and andromeda search engine integrated into the MaxQuant environment (versions 1.5.2.8)^72^ for label-free quantification (LFQ) of proteins, and searched against a non-redundant UniProt human database (20,325 sequences) using default settings with the following minor changes. The parameters were set as follows: trypsin enzyme, maximum two missed cleavages, precursor-ion mass tolerance was set to 10 ppm; fragment-ion mass tolerance was set to 0.05 Da, carbamidomethyl (C) as static modification and several dynamic modifications were oxidation (M). In addition, thioacylation (K and protein N-term) and CAMthiopropanoyl (K and protein N-term) were also set as dynamic modifications for protein identification. The matching time window of “match between runs” was set as 0.7 min, and the parameters of “Min. peptides”, “Min rator + unique peptides” and “Min. unique peptides” were set as 2, 2, 1 for protein quantification, respectively. The FDR was controlled < 1% in every search result.

#### Bioinformatic analysis

Protein cellular localization and statistical enrichment test were analyzed by PANTHER software (http://pantherdb.org/)^73^ and UniProt database (http://www.uniprot.org).

To compare the surface proteins between BPH and tumor samples, LFQ intensities for proteins without missing values or two missing values in both runs were first averaged, or the unique LFQ intensity for proteins with one missing value was directly retained in each sample. The method of analyzing the differential expression of surface proteins between BPH and tumor samples is the same as the method of analysis of proteomic data.

### High-throughput drug screening

Thirty-three FDA-approved drugs were selected for drug screening (Supplementary Data 5). The majority of medication stocks (10 mM in DMSO, excluding platinum compounds) were kept at −80 °C. Due to the fact that DMSO might deactivate platinum complexes, cisplatin, carboplatin, and nedaplatin were dissolved in 20 mM PBS. To assure that each primary cell line was in the growth phase at the termination of the assay (85% confluency), we seeded cells in 384-well microplates at 15% confluency and estimated the ideal cell number for each cell line based on cell proliferation rate (Supplementary Fig. 7a). During screening, the cells were grown in the primary medium at 5% CO_2_ and 37 °C. The pharmaceutical screening was performed on high-throughput drug screening platform in our institute.

Cells were seeded in 384-well plates at a predetermined cell density and volume of 50 μL using a Multidrop Combi Reagent Dispenser for drug screening (Thermo Fisher Scientific). After an overnight incubation, cells were treated using JANUS (PerkinElmer) with 10-fold serial dilutions of seven doses of a single drug and then transferred to an incubator for 72 h.^9,10,16^ Upon completion of drug treatment, each well was added 25 μL CellTiter-Glo reagent (Promega), and after 10 min incubation at room temperature, the luminescent signal was evaluated using EnSpire Multilabel Reader (PerkinElmer) to quantify cell viabilities. Every treatment was carried out in triplicate wells. On every screening plate, a high single-point concentration of Bortezomib that caused total cell death was employed as a positive control.

Multi-parametric analysis of drug–response curves of IC_50_ and activity area (AA) was performed using Python. IC_50_ assesses drug potency and efficacy,^74^ whereas AA represents drug–response magnitude.^11^ The IC_50_ and AA values can be used to describe the drug reaction and offer information about various elements.

### mRNA-protein correlation

Gene-wise correlation was used for the correlation assessment of RNA-Seq and TMT proteomics platforms.^75^ Spearman’s correlation was calculated for each gene across all the samples. Gene without missing values and with top 10% SD in RNA-Seq and TMT datasets were chosen for analysis.

### Pathway enrichment analysis

Gene set enrichment analysis (GSEA) software was used for pathway enrichment (KEGG and Hallmark, MSigDB v7.1.).^69^ The permutation type was set as “gene-set”. For proteomic and surface proteomic datasets, proteins (gene level) with less than 20% of missing values were selected, the missing values were then imputed via KNN method using R package “impute”.^36^ Pathway with FDR less than 0.05 was used.

### Pharmacoproteomic analysis in PCMR

Correlation between drug sensitivity and proteome expression level was evaluated by Spearman correlation coefficients between 7-AA values and normalized protein intensities. Proteins with less than 50% missing value across all samples were preserved in the analysis. Drug/proteins pairs with a cutoff of *p*-value <0.05 were considered as significantly associated, otherwise, the correlation coefficients were reset to missing values. For each drug, the number of CRGs positively or negatively associated with the drug (*p*-value < 0.05) was calculated respectively.

### Experimental validation of gene functions

PEI reagent was used to deliver the pGREEN plasmids containing shAGR2 into 293T cells along with the plasmids psPAX2 and pMD2.G (Addgene) for lentivirus packaging. The lentivirus-containing supernatant was collected on the second and third day after transfection, then filtered with 0.45 μm syringe filters (PALL Life Sciences). Lentiviruses were extracted from the supernatant and kept at −80 °C until use. Infecting 1 × 10^5^ PC3 cells in a 6-well format with 2 μg/ml polybrene (Sigma) using viral supernatant, then the cells were centrifuged at 1500 rpm for 2 h.

Western blotting was used to evaluate the knockdown effectiveness. For 6-well format siRNA transfection, 2.5 μL siRNA in 250 μL serum-free opti-MEM medium (Gibco, 31985070) was combined with 4 μL RNAiMax (Invitrogen) in an equivalent volume of serum-free opti-MEM media. After 20 minutes, the siRNA-lipid mixture was transferred to a 6-well plate and 5 × 10^5^ cells were seeded per well. After 48 h, the transfected cells were treated with the drug at the specified dosages and times. In this investigation, pairs of AGR2 primers were presented in Supplementary Table 2.

### Western blot assay and antibodies

Using RIPA lysis buffer supplemented with PMSF and Protease Inhibitor Cocktail, primary cells were lysed on ice and quantified using the BCA Protein Assay Kit. 20 μg protein of cell lysates were separated using 10% SDS-polyacrylamide gel electrophoresis (SDS-PAGE) and transferred to a nitrocellulose (NC) membrane (Pall). After blocking membranes with 10% non-fat dried milk in phosphate-buffered saline and 0.1% Tween 20 solution, the following primary antibodies were incubated overnight at 4 °C with the membranes: Phospho-AKT (CST, 4060), AKT (CST, 4691), Phospho-c-MET (CST, 3077), c-MET (CST, 3127), Phospho-ALK (CST, 3983), ALK (CST, 3633), AMACR (Abcam, ab246927), AR (Abcam, ab268062), CK5 (Abcam, ab52635), E-cadherin (HuaAn, EM0502), Vimentin (HuaAn, EM0401), AGR2 (Proteintech, 12275-1-AP), Tubulin (Sigma-Aldrich, T6793), GAPDH (Proteintech, 10494-1-AP). The membranes were then incubated with the appropriate secondary antibody for 6 h at 4 °C. Target proteins were visualized using the EZ ECL pico luminescence reagent (Life-iLab, AP34L025) with the ChemiScope S6 system (Clinx, China). Relative expression was normalized to GAPDH using the ImageJ software (Version 1.52q, National Institutes of Health, MD, USA).

To evaluate the efficiency of biotin labeling, 20 μg of labeled cell lysates via EZ-Link^TM^ Sulfo-NHS-SS-biotin were re-dissolved in non-reducing SDS-PAGE sample buffer and then run on two SDS-PAGE. One was stained by Coomassie Brilliant Blue method as protein level control and the other was transferred onto a PVDF membrane (0.45 μm, Millipore). Subsequently, the membrane was treated overnight at 4 °C with HRP-labeled streptavidin (CST, 3999) diluted 1:4000 in blocking buffer. ChemiScope S6 system was used to visualize the target proteins.

To confirm the protein abundance of AGR2 on the cell surface and whole cell lysate across BPH and prostate primary cell samples, 100 μg of labeled primary cell samples via EZ-Link^TM^ Sulfo-NHS-SS-biotin were first enriched by affinity purification, followed by pulldown with non-reducing SDS-PAGE sample buffer. Simultaneously, 20 μg of total cell lysates for each sample were boiled with non-reducing SDS-PAGE sample buffer. The enriched surface fraction and total cell lysates were then run on SDS-PAGE, transferred onto a NC membrane, and blocked, respectively. The membranes were then incubated with AGR2 or GAPDH overnight at 4 °C. Target proteins were visualized using the EZ ECL pico luminescence reagent with the ChemiScope S6 system.

### Statistics

For statistics, Python(3.6), R (3.6.1) and GraphPad (8.4.0) were utilized. When applicable, the Fisher’s exact test or Wilcoxon rank-sum test was used to investigate group differences. Unless otherwise specified, all examinations were two sided. The survivals of groups were compared using the log-rank test. Cox proportional hazard model was utilized to calculate HR and its 95% confidence interval.

## Supplementary information

SUPPLEMENTAL MATERIAL

Dataset 1

Dataset 2

Dataset 3

Dataset 4

Dataset 5

Dataset 6

Supplementary Table S1

Supplementary Table S2

## Data Availability

The MaxQuant result files, fasta database and original proteomics raw data generated or downloaded in this study has been uploaded to the iProX Consortium with the subproject ID IPX0002297000 (URL: https://www.iprox.cn/page/PSV023.html;?url=1643030858545u jxN, Password: XGpC). The MaxQuant and PD result files, original raw data of surface proteomics was uploaded onto JPOST Repository^76^. The accession numbers are PXD031476 for ProteomeXchange and JPST001448 for jPOST (URL: https://repository.jpostdb.org/preview/484570237620121d df1960; Access key: 3319). The RNA-Seq data and WES data reported in this paper have been deposited in the Genome Sequence^77^ in National Genomics Data Center^78^, China National Center for Bioinformation / Beijing Institute of Genomics, Chinese Academy of Sciences (GSA-Human: HRA002706) that are publicly accessible at https://ngdc.cncb.ac.cn/gsa-human. Detailed results of RNA-Seq data and WES were included in Supplementary Data 1 and Supplementary Data 2.
