# Supplementary material for: Integrative multi-omics and drug–response characterization of patient-derived prostate cancer primary cells
Source: Signal Transduct Target Ther. 2023 May 1;8:175. doi: 10.1038/s41392-023-01393-9 (PMC10149505; doi:10.1038/s41392-023-01393-9)

**Supplementary Materials**  **for**

**Integrative multi-omics and drug response characterization of patient-derived prostate cancer primary cells**

**Authors**

Ziruoyu Wang^1,9^, Yanan Li^3,9^, Wensi Zhao^4,7,9^, Shuai Jiang^5,8,9^, Yuqi Huang^4,7^, Jun Hou^5^, Xuelu Zhang^2^, Zhaoyu Zhai^2^, Chen Yang^6^, Jiaqi Wang^1^, Jiying Zhu^1^, Jianbo Pan^2^, Wei Jiang^1^, Zengxia Li^1^, Mingliang Ye^3,*^, Minjia Tan^4,*^, Haowen Jiang^6,*^ & Yongjun Dang^2,1,*^

**Affiliations**

^1^Key Laboratory of Metabolism and Molecular Medicine, The Ministry of Education, Department of Biochemistry and Molecular Biology, School of Basic Medical Sciences, Shanghai Medical College, Fudan University, Shanghai, 200032, China. ^2^Center for Novel Target and Therapeutic Intervention, Chongqing Medical University, Chongqing, 400016, China. ^3^CAS Key Lab of Separation Sciences for Analytical Chemistry, National Chromatographic Research and Analysis Center, Dalian Institute of Chemical Physics, Chinese Academy of Sciences, Dalian, 116023, China. ^4^The Chemical Proteomics Center and State Key Laboratory of Drug Research, Shanghai Institute of Materia Medica, Chinese Academy of Sciences, Shanghai, 201203, China. ^5^Department of Urology, Zhongshan Hospital, Fudan University, Shanghai, 200032, China. ^6^Department of Urology, Huashan Hospital, Fudan University, Shanghai, 200040, China. ^7^University of Chinese Academy of Sciences, Beijing, 100049, China. ^8^Department of Urology, Zhongshan Hospital Wusong Branch, Fudan University, Shanghai, 200032, China. ^9^These authors contributed equally: Ziruoyu Wang, Yanan Li, Wensi Zhao, Shuai Jiang. *Corresponding authors

Correspondence to: [ziruoyu.wang@gmail.com](mailto:ziruoyu.wang@gmail.com) and [yongjundang@fudan.edu.cn](mailto:yongjundang@fudan.edu.cn)

This PDF file includes:

supplementary figures 1-10.

**Supplementary Figure 1. Characteristic validation of PCa primary cell lines.
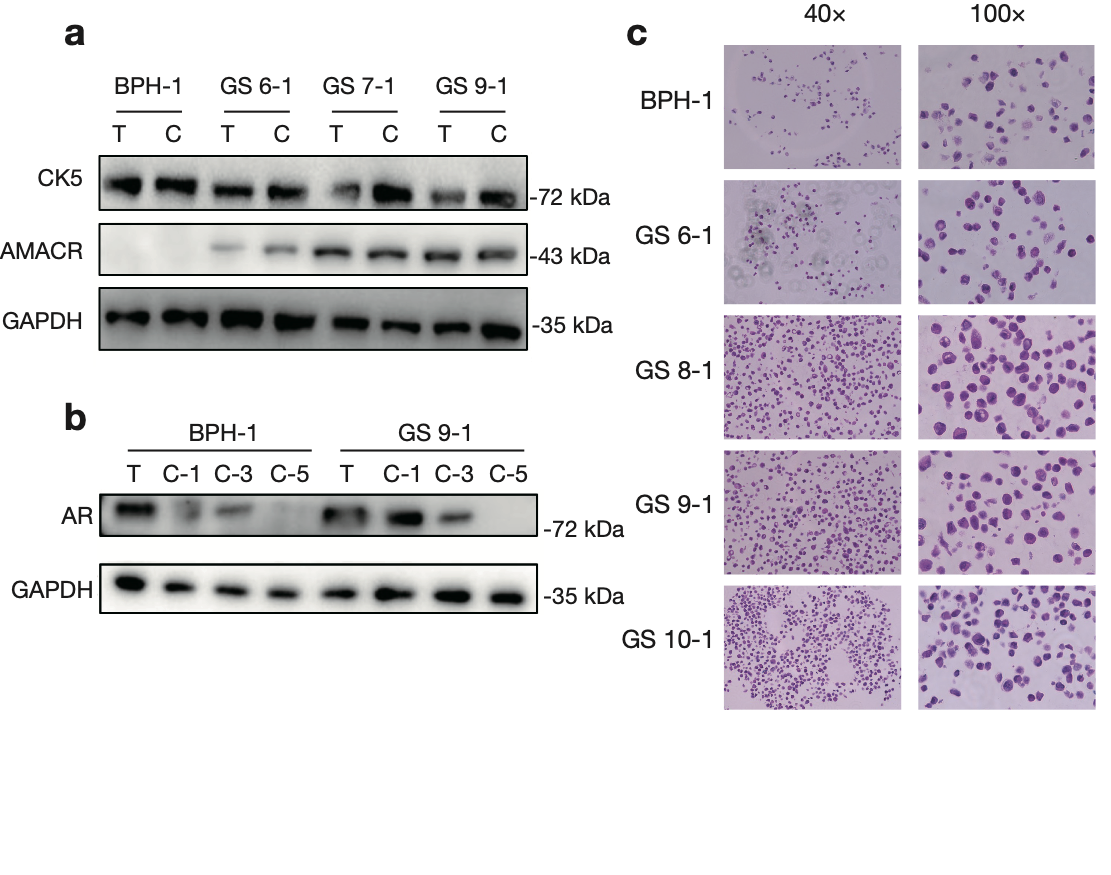
**

**(a)** Western blot analysis of CK5 and AMACR in tissues (T) and corresponding primary cells (C). **(b)** Immunoblotting of AR expression in BPH- and tumor-derived primary cells in different passages. **(c)** HE staining of primary cells derived from prostate tissues with different degrees of malignancy.

*
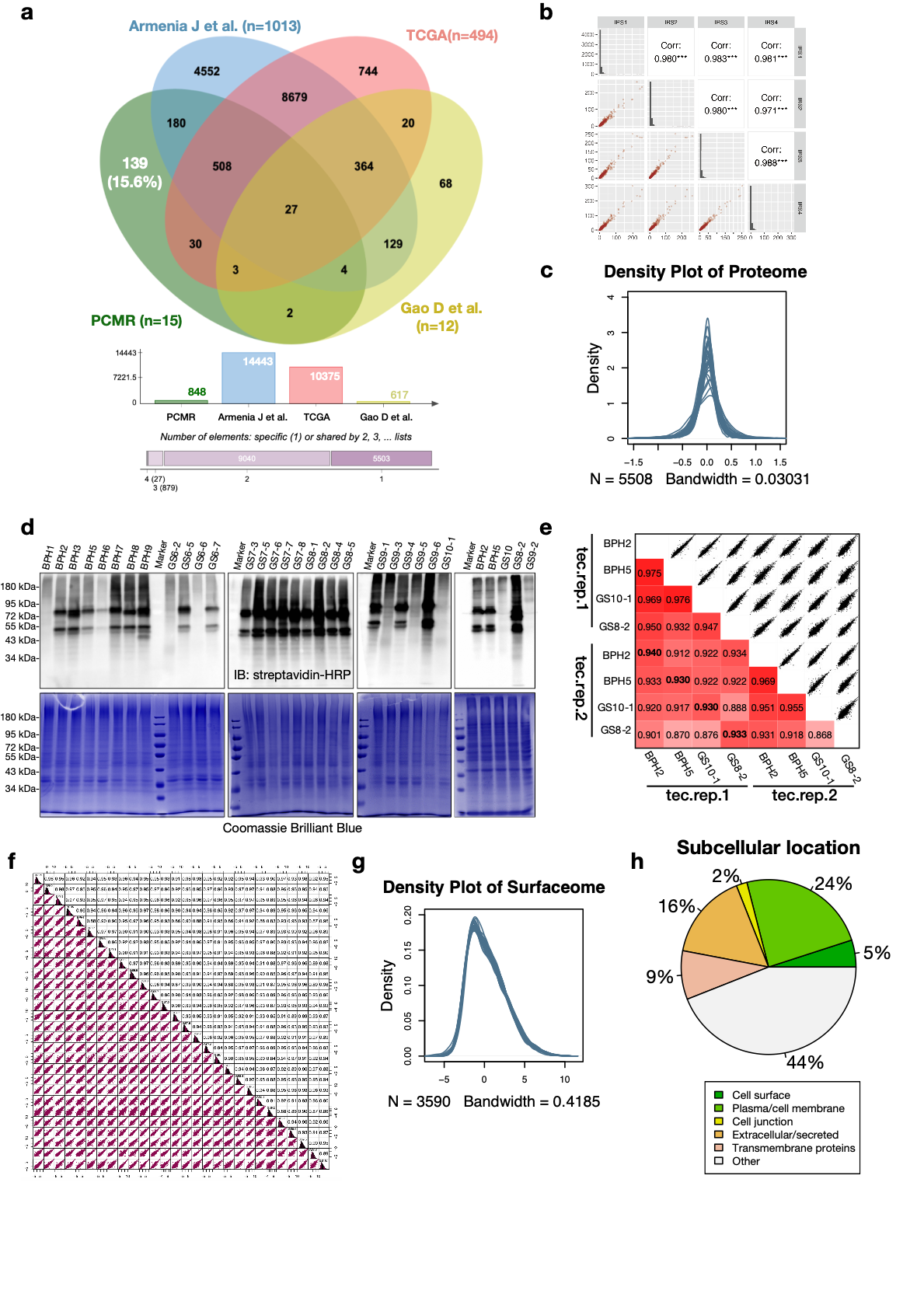
*

**Supplementary Figure 2. Multi-omics landscape and quality control of PCa primary cell lines.**

**(a)** Venn diagram showing the overlapped mutations in PCMR and previous prostate cancer sequencing studies in cBioPortal. **(b)** Correlation analysis of the mix of cell samples as MS quality control to evaluate the robustness of TMT quantification. Top-right half panel: pairwise calculation of Pearson’s correlation coefficients; bottom-left half panel: pairwise comparison by scatterplots. **(c)** Samples distribution of protein abundances by a density plot. A unimodal distribution (dip test) was observed. **(d)** Biotinylation of individual primary cell samples was analyzed by streptavidin-blot. The gel was stained by Coomassie Brilliant Blue method as protein level control. **(e)** Correlation analysis of four replicated primary cell samples as MS quality control to evaluate the robustness of label-free quantification. **(f)** Correlation analysis of surface protein abundances of each primary cell samples as MS quality control to evaluate the robustness of label-free quantification. **(g)** Samples distribution of surface protein abundances by a density plot. A unimodal distribution (dip test) was observed. **(h)** Pie chart showing protein subcellular location of cell-surface proteome data was analyzed by GO-annotation of cell surface/plasma membrane/cell membrane/extracellular and transmembrane topology prediction using TMHMM and Phobius.


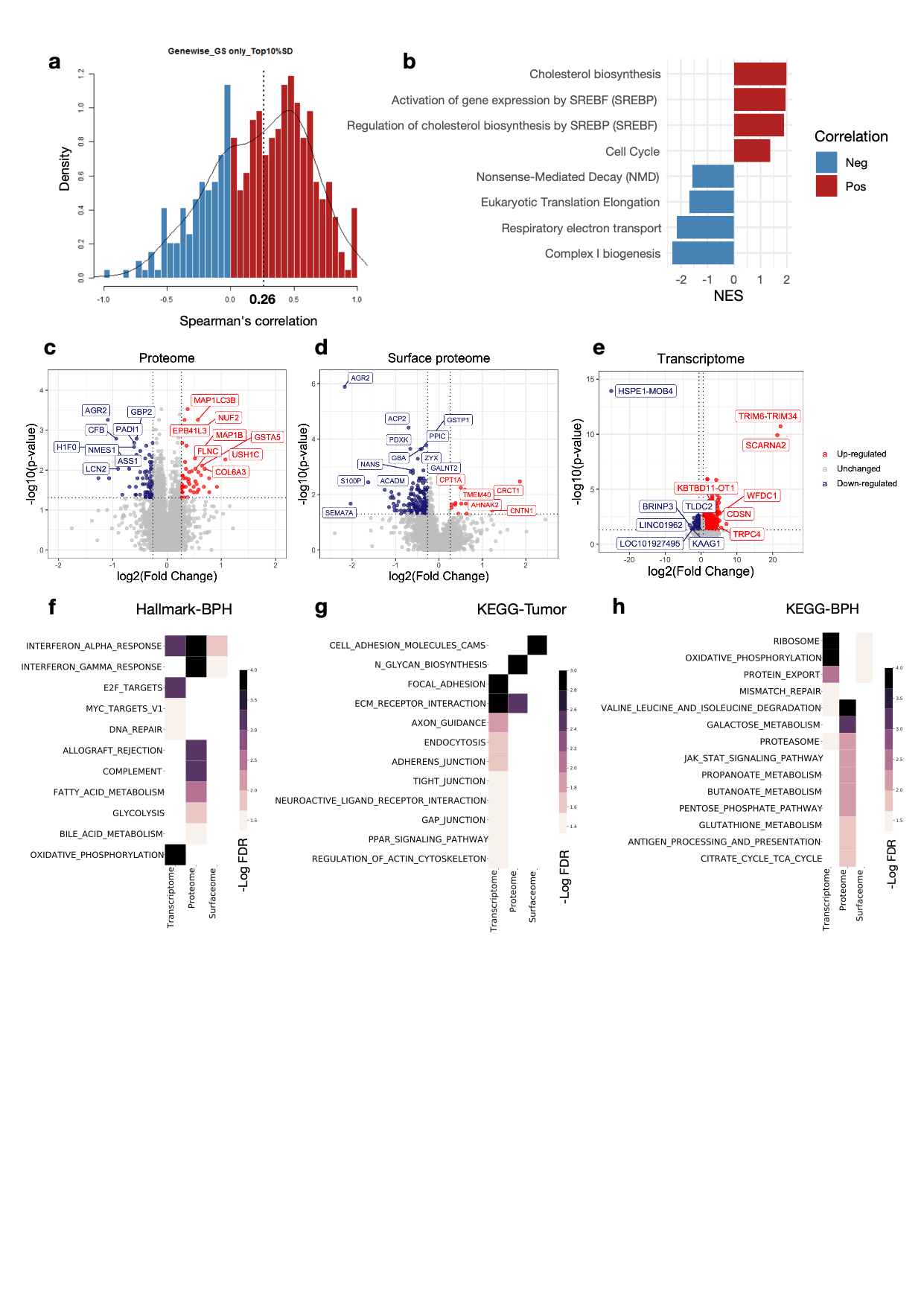


**Supplementary Figure 3. Mutation OncoPrint of top 10 mutated genes.
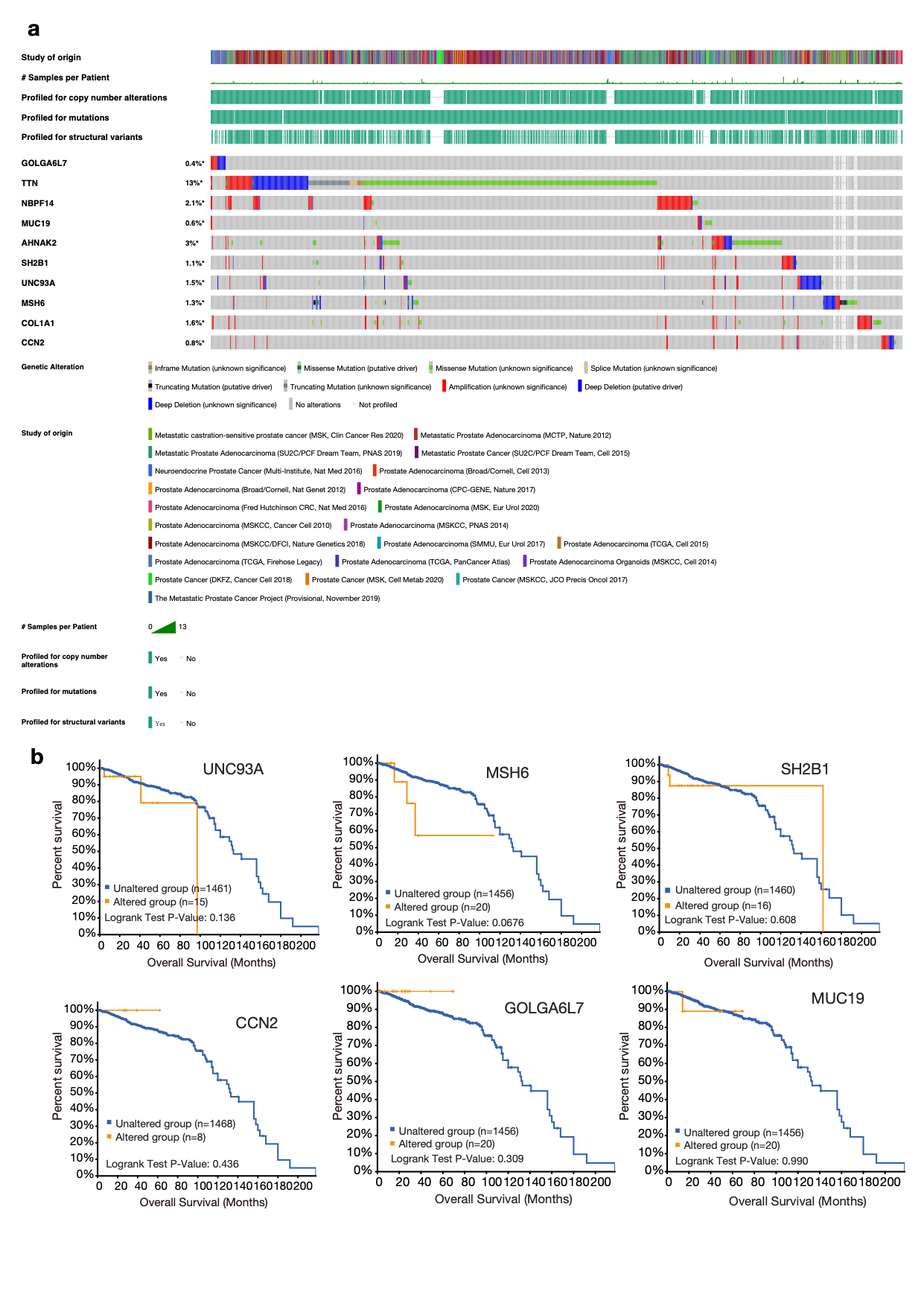
**

**(a)** cBioPortal OncoPrint evaluate the mutations attributes of top 10 mutated genes in primary cells. **(b)** The survival curve of six prostate cancer related genes (UNC93A, MSH6, SH2B1, CCN2, GOLGA6L7, MUC19) that indicate less prognostic value. Data from 7308 samples of 22 previous studies collected and summarized in cBioPortal.

**Supplementary Figure 4. Integrative analyses of transcriptomic, proteomic, and surface proteomic data in PCa primary samples.**

**(a)** mRNA-protein correlation in tumor samples. **(b)** Pathways involved positively or negatively correlated in mRNA and protein levels using GESA method (FDR < 0.05). **(c-e)** Differentially expressed genes identified in proteome, surface proteome and transcriptome. **(f-h)** Enrichment pathways revealed by GSEA (hallmark and KEGG gene set) analysis in BPH or tumor patients (*p*-value from log rank test).
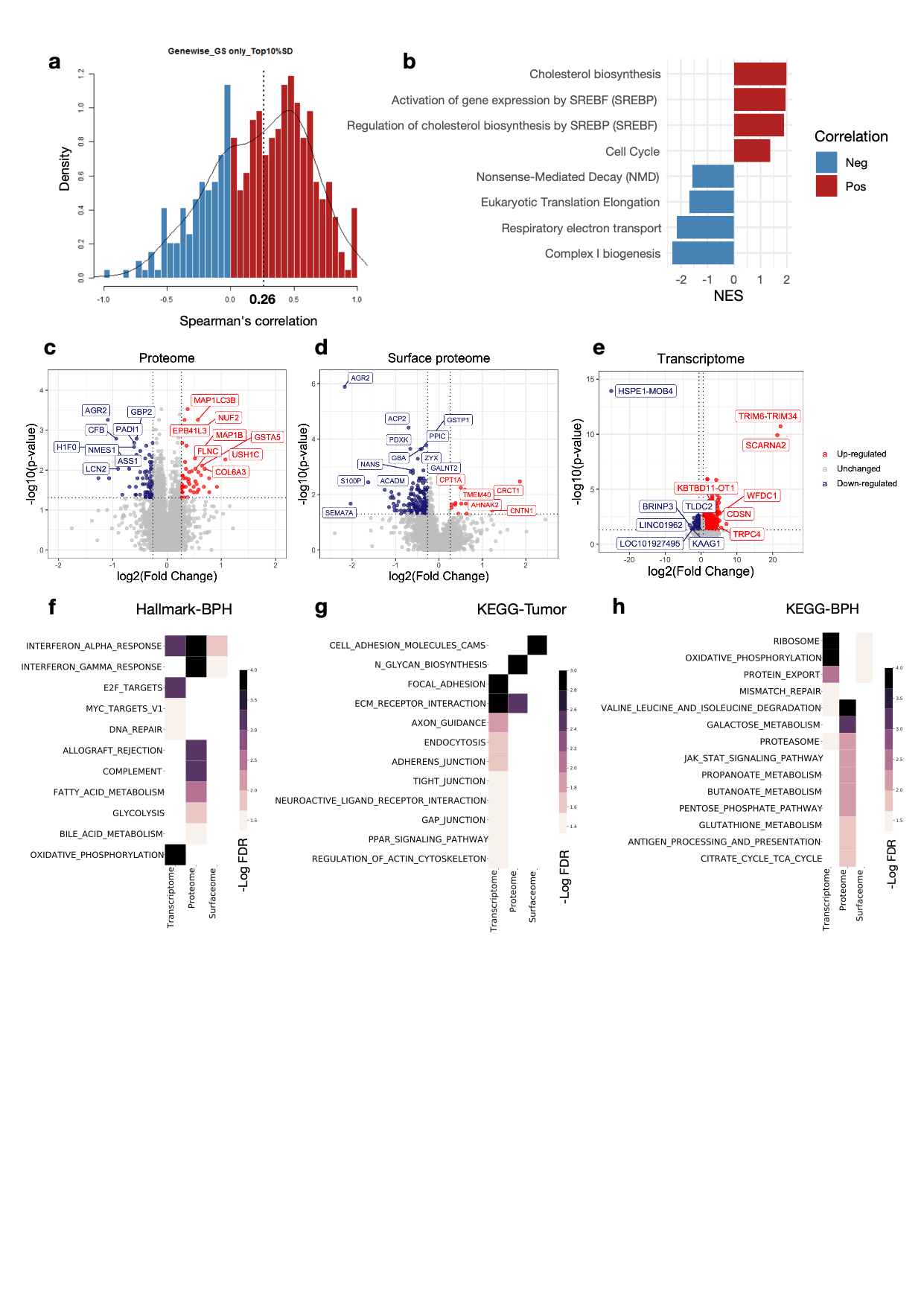


**
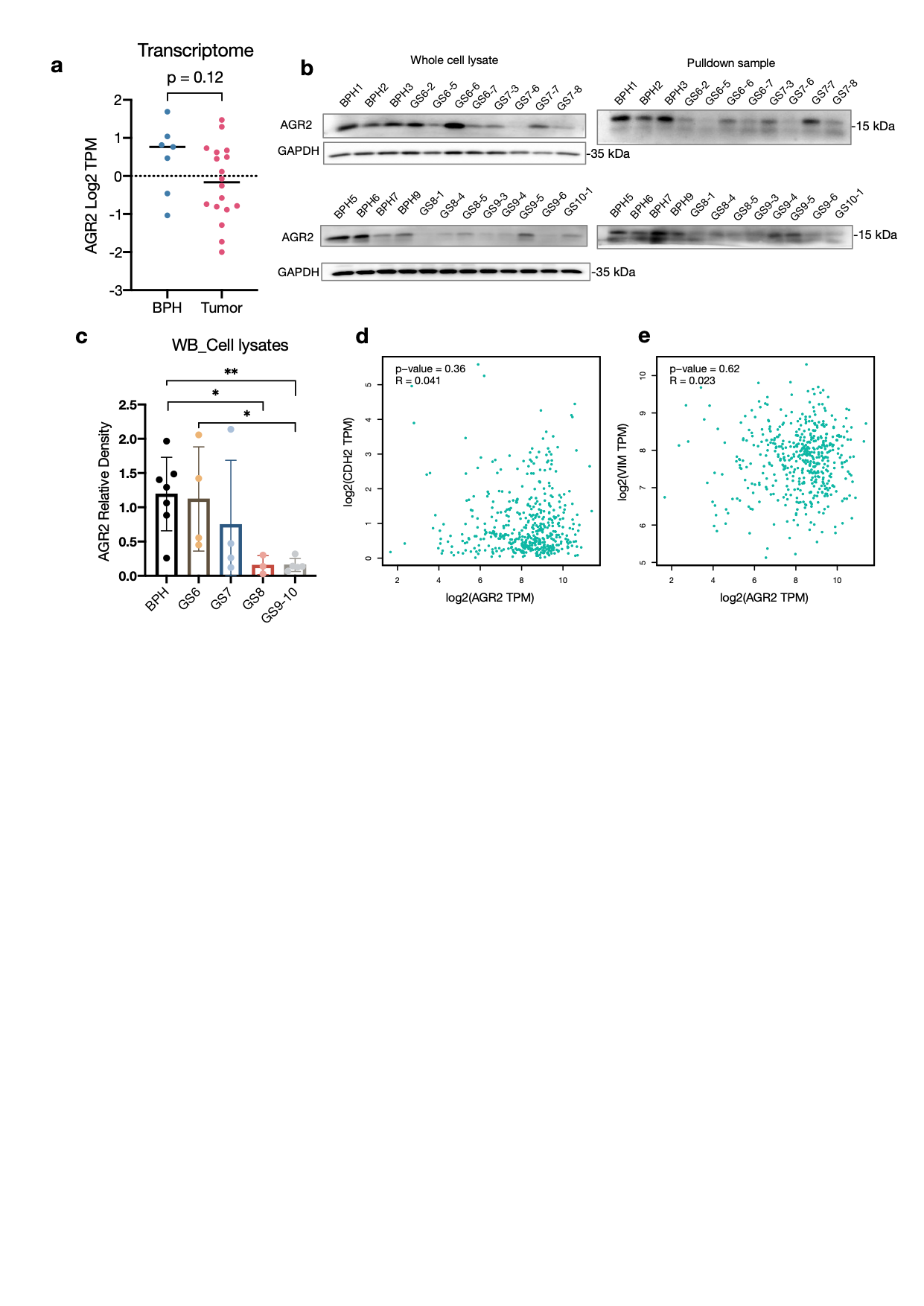
Supplementary Figure 5. AGR2 expression level identification.**

**(a)** AGR2 mRNA expression levels in tumor and BPH primary cells evaluated by log2 (TPM) level. **(b)** Representative western blot analysis of AGR2 in protein (whole cell lysate sample) and surface protein (pulldown sample) levels of 23 independent cell samples collected from tumor and BPH patients. **(c)** Quantification of the whole cell lysate western blotting data (*p < 0.05, **p < 0.01 by Wilcoxon rank-sum test). **(d, e)** Scatter plots showing the Spearman’s correlation between AGR2 and 2 mesenchymal-related genes (CDH2, VIM) in PRAD (n = 494) among TCGA databa

**Supplementary Figure 6. Disease-free survival analysis (DFS) of different AGR2
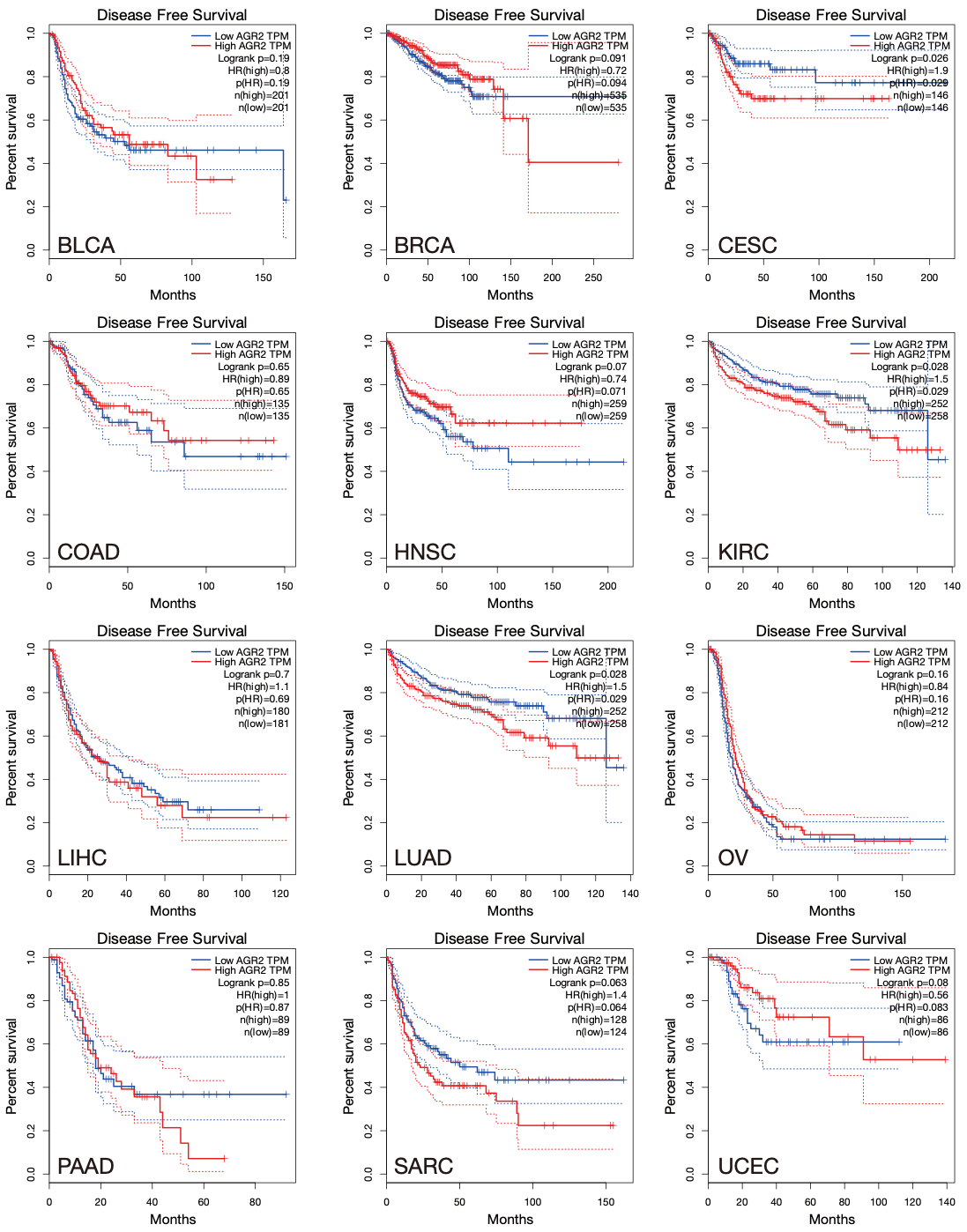
 expression levels in various TCGA datasets.**

DFS promoted by GEPIA. Blue, low AGR2 mRNA expressed samples. Red, high AGR2 mRNA expressed samples. BLCA, bladder urothelial carcinoma; BRCA, breast invasive carcinoma; CESC, cervical squamous cell carcinoma and endocervical adenocarcinoma; COAD, colon adenocarcinoma; HNSC, head and neck squamous cell carcinoma; KIRC, kidney renal clear cell carcinoma; LIHC, liver hepatocellular carcinoma; LUAD, lung adenocarcinoma; OV, ovarian serous cystadenocarcinoma; PAAD, pancreatic adenocarcinoma; SARC, sarcoma; UCEC, uterine corpus endometrial carcinoma (*p*-value from log-rank test).

**Supplementary Figure 7. Drug response data and whole-wide
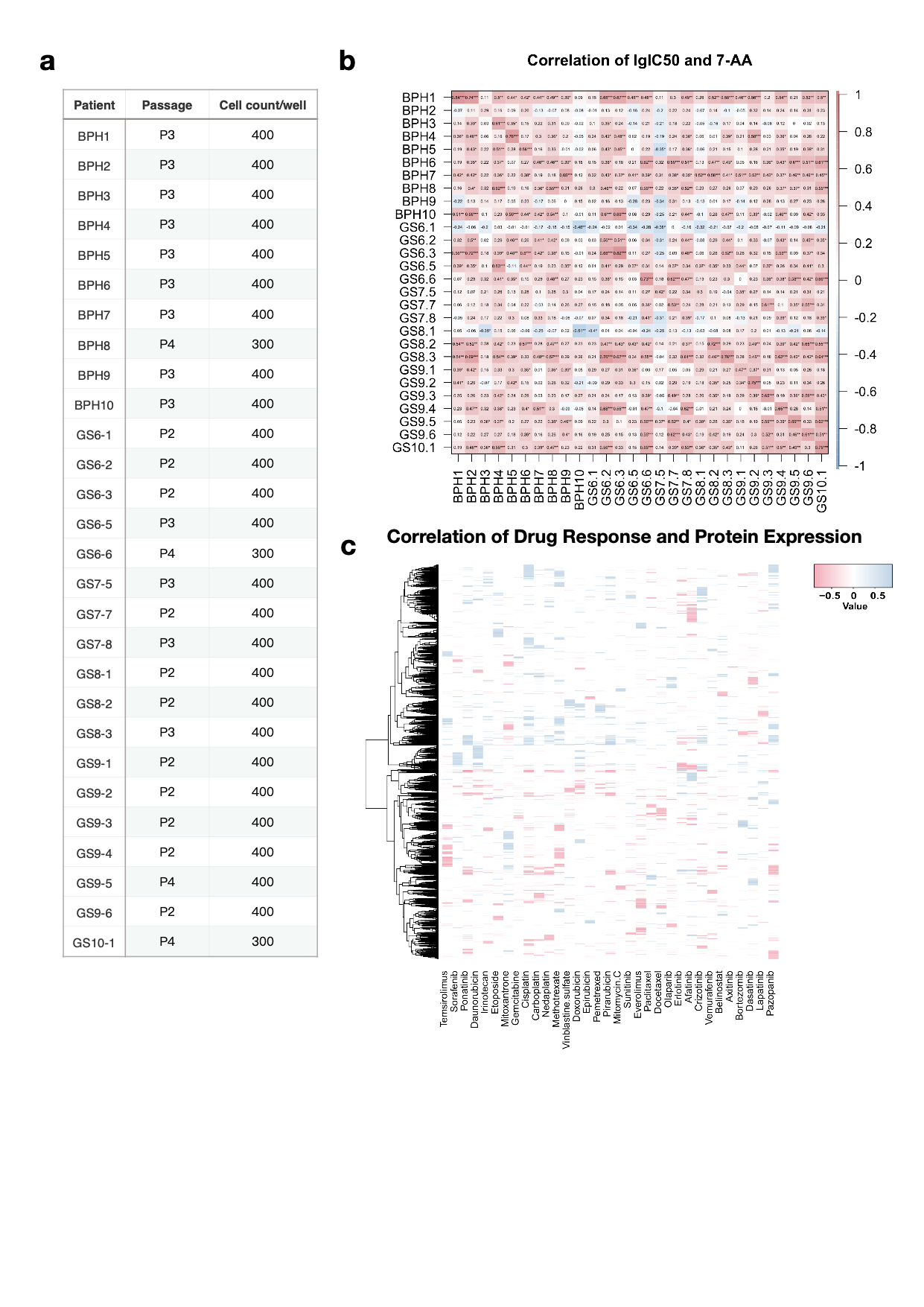
 pharmacoprote-omic analysis.**

**(a)** The specific details regarding each cell line employed in the drug screening. Passage, the precise primary cell passage number; Cell count/well, the number of cells seeded in each 384-well. **(b)** Spearman’s correlation between lg (IC50) and 7-activity area (7-AA). Blue, low correlation; red, high correlation (*p < 0.05, **p < 0.01, ***p < 0.001, ****p < 0.0001). **(c)** Spearman’s correlation between 7-AA and protein expression levels. Blue, low correlated pairs; red, high correlated pairs.

**Supplementary Figure 8. Correlations between Crizotinib response and CRGs expression.**

The Spearman’s correlation between Crizotinib 7-AA levels and CRG expression levels.


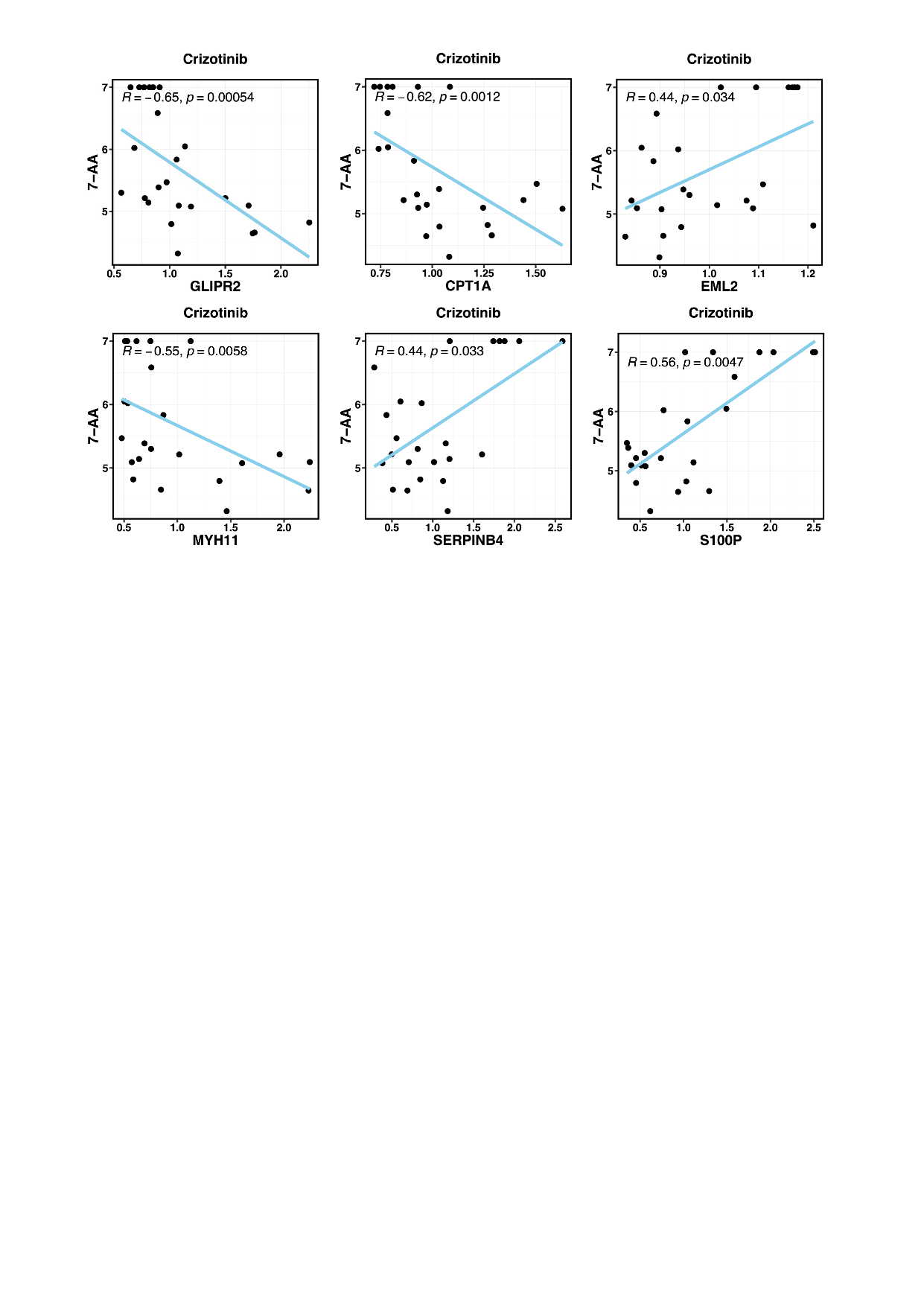


**Supplementary Figure 9. AGR2 knockdown effect in PC3 model.
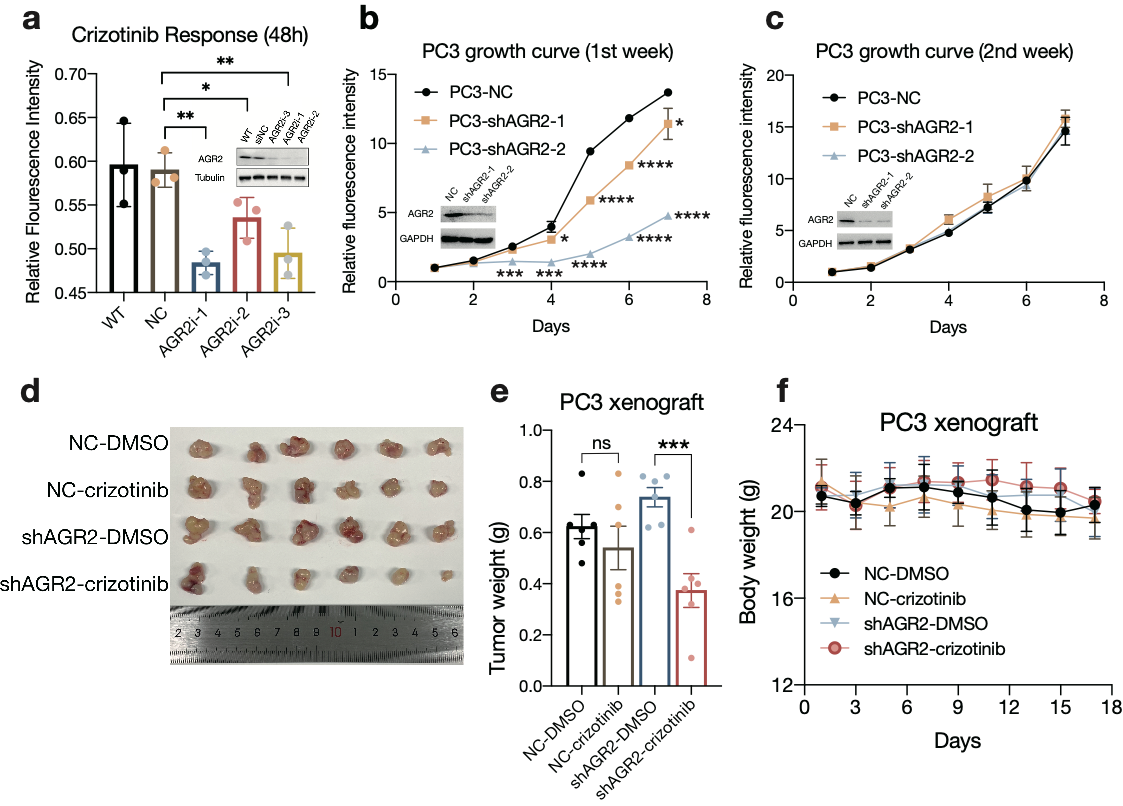
**

**(a)** The relative fluorescence intensity of PC3-WT/NC/siAGR2 cell viability measured by CellTiter Glo after treatment with 10 μM crizotinib for 48h. The data was presented as mean ± SD, n=3. The fluorescence intensities were standardized to the DMSO treatment of each cell line. WB analysis of AGR2 knockdown effect is shown in figure. Experiments were biologically repeated in triplicate, and one representative result is shown. **(b-c)** Growth curve and western blot analysis after NC/shAGR2 lentiviral infection of PC3 cells in the first week (b) and the second week (c). The data was presented as mean ±  SD, n = 3. WB analysis of AGR2 knockdown effect is shown in figure. Experiments were biologically repeated in triplicate, and one representative result is shown. **(d-e)** Gross anatomy (c) and tumor weight (d) of final dissected xenograft tumor masses. The data was presented as mean ± SEM, n = 6 mice/group. **(f)** Body weight measurement of nude mouse xenografted PC3 cells with/without AGR2 knockdown during the 18 days with 25 mg/kg crizotinib treatment. The data was presented as mean ± SD, n = 6 mice/group.

*p < 0.05, **p < 0.01, ***p < 0.001, ****p < 0.0001 by unpaired two-sided Student’s t test.

**Supplementary Figure 10. Low AGR2 expression stimulates RTK signaling.**

**(a-b)** Principal-component analyses (PCAs) of RNA-seq (a) and proteomics data (b) in three BPH and three tumor primary cells which showed the most considerably different crizotinib susceptibility. **(c-e)** GSEA pathway enrichment analyses using GO (c), KEGG (d) and Reactome (e) database revealed pathways that were altered in three tumor cells. The abscissa represents the log2(fold change) of genes in certain pathway. **(f)** Normalized inhibition levels of MK-2206 and crizotinib in heatmap. **(g)** The drug-drug interaction evaluated for PC3-shAGR2 cell line treated with the drug combination MK-2206/crizotinib for 72 h. The model used for the calculation of Log CI of the dose–response curve is the log-logistic with three parameters (log-logistic[01]) from the data shown in (f), and for the calculation of the CI of the Loewe model.
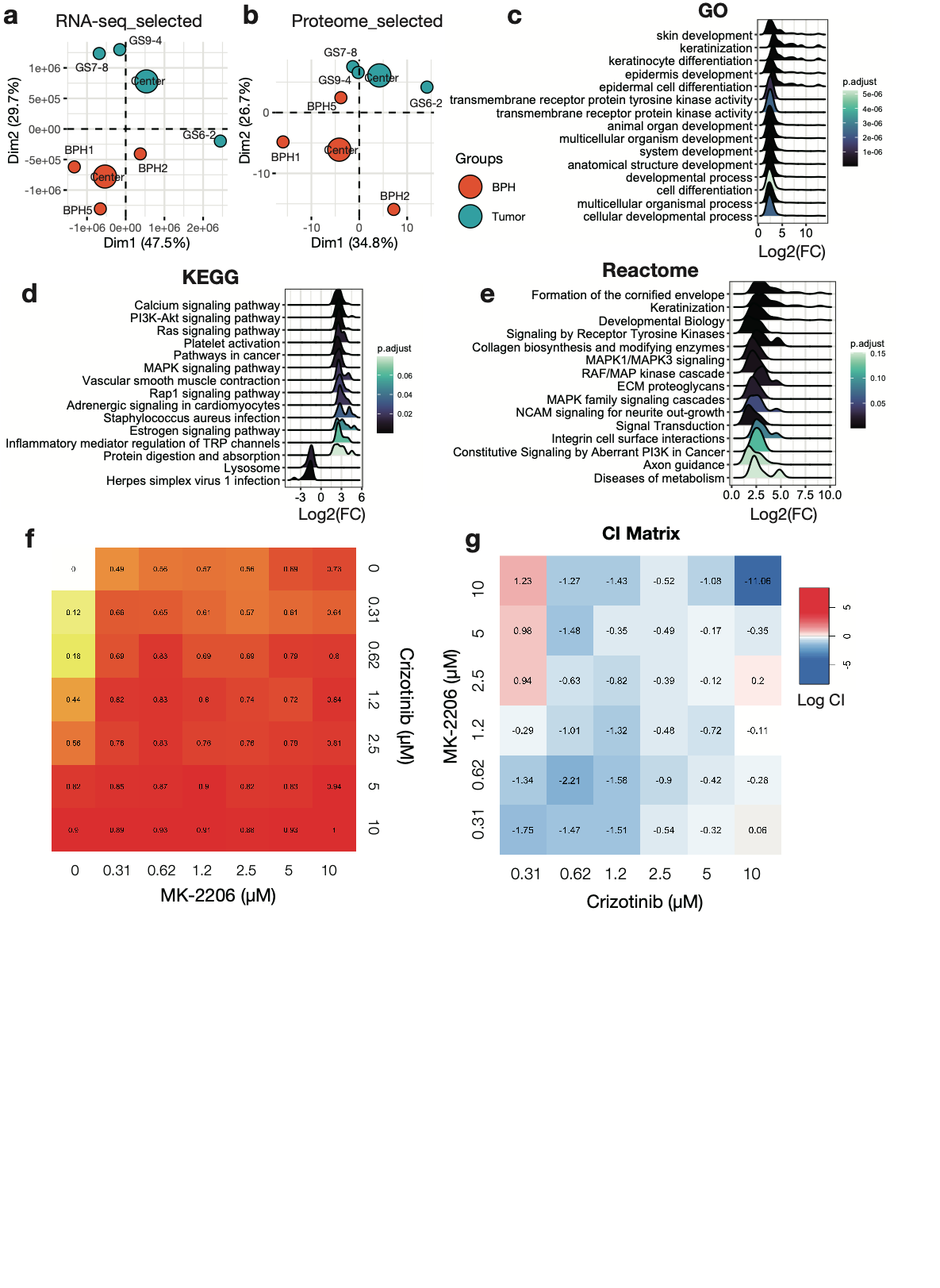

Supplement: Supplementary file 1 — SUPPLEMENTAL MATERIAL [file 41392_2023_1393_MOESM1_ESM.docx]
